# 2D Laminated Hybrid Architectures Based on Graphene/Mesoporous Polyaniline for High‐Performance Supercapacitor Electrode

**DOI:** 10.1002/smll.202600004

**Published:** 2026-03-31

**Authors:** Jiashu Li, Chenchen Zhang, Hongyi Zhang, Liangyu Dong, Bin Han, Leijing Liu, Shaohua Liu, Zhaoyang Liu, Wenjing Tian, Paolo Samorì

**Affiliations:** ^1^ State Key Laboratory of Supramolecular Structure and Materials Jilin University Changchun China; ^2^ ISIS & icFRC University of Strasbourg & CNRS Strasbourg France; ^3^ State Key Laboratory of Precision Spectroscopy Engineering Research Center of Nanophotonics & Advanced Instrument (Ministry of Education) School of Physics and Electronic Science East China Normal University Shanghai China

**Keywords:** 2D materials, graphene, mesoporous polyaniline, supercapacitor

## Abstract

Graphene has emerged as a highly promising candidate for high‐performance supercapacitor electrodes. Its atomically‐thin 2D structure and exceptional physical properties provide an ideal foundational platform for designing hybrid nanomaterials, which are pivotal for advancing electrochemical performance metrics. However, the practical use of these graphene‐based hybrids faces key challenges, including the degradation of electronic properties due to defects introduced during synthesis, and the insufficient control over the loading and distribution of pseudocapacitive components, which compromises their synergistic potential. To address these fundamental issues, we developed a novel 2D laminated hybrid architecture by using electrochemically exfoliated graphene (EG) and mesoporous polyaniline (PANI) nanosheets, synergistically combining electrical double‐layer capacitance and pseudocapacitance. A high‐shear mixing technique ensures uniform nanoscale blending at various ratios while preventing graphene restacking. Strong interfacial interactions confirm the formation of a laminated hybrid architecture, leading to an optimized microstructure with significantly enhanced charge storage characteristics. The hybrid electrode achieves a remarkable volumetric capacitance of 424.58 F/cm^3^ and superior rate capability. Our work provides a rational design approach for the construction of graphene‐based 2D laminated hybrid architecture, advancing next‐generation energy storage technologies.

## Graphical abstract

We demonstrate a rational and scalable strategy for the construction of 2D hybrid architecture by the liquid‐phase co‐assembly of graphene and polyaniline nanosheets. A high‐shear mixing process enables face‐to‐face stacking, creating a robust interface with strong electronic coupling. The resulting electrode delivers a high volumetric capacitance and superior rate capability, outperforming most advanced graphene‐based supercapacitors.

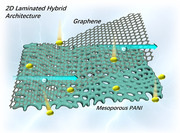

## 1 Introduction

The escalating global energy crisis has driven urgent demand for sustainable energy storage solutions [1, 2, 3]. Electrochemical supercapacitors have emerged as leading candidates in this pursuit, offering an exceptional combination of high‐power density, ultra‐long cycle stability, inherent safety, and low maintenance costs [4]. These characteristics position supercapacitors as the pivotal energy storage platform bridging the performance gap between conventional capacitors and batteries.

As a performance‐determining component of supercapacitors, advanced electrode materials must combine high charge storage capacity with rapid kinetics and stability. Among carbon‐based nanomaterials, 2D graphene has emerged as the premier candidate due to its unparalleled properties combination, which includes an ultrahigh theoretical surface area (2630 m^2^/g), exceptional conductivity (∼10^6^ S/m), and robust electrochemical stability [5, 6, 7, 8, 9, 10]. These characteristics directly address the power‐density and cyclability demands of next‐generation supercapacitors. The electrochemical performance of graphene in supercapacitors is intrinsically linked to its production method. High‐temperature chemical vapor deposition (CVD), which produces high‐quality films but suffers from limited scalability and transfer‐related contaminants [11, 12, 13, 14]. Conventional graphene oxide reduction routes, while enabling solution processability, inevitably introduce oxygenated defects and structural imperfections that degrade conductivity, ultimately compromising performance [15, 16, 17, 18, 19, 20, 21]. In contrast, electrochemical exfoliation of graphite preserves the pristine lattice structure of graphene while maintaining solution processability, as it avoids aggressive oxidation and minimizes structural damage [22, 23, 24, 25]. By precisely controlling parameters such as applied potential, electrolyte composition, and intercalation time, this approach enables tunable flake thickness while retaining graphene's intrinsic high mobility, which is a critical advantage for high‐performance electrodes [24, 25, 26]. This balance of quality, scalability, and processability makes electrochemically exfoliated graphene (EG) uniquely suited for high‐performance supercapacitor applications where both conductivity and accessible surface area are paramount [27, 28, 29].

On the other hand, the performance of graphene‐based supercapacitors is fundamentally constrained by their exclusive dependence on electric double‐layer capacitance (EDLC), which inherently lacks the enhanced charge storage capabilities of faradaic pseudocapacitance. Extensive efforts have focused on developing high‐performance pseudocapacitive electrode materials to enhance supercapacitor performance, including metal oxides (e.g., RuO_2_ [30], MnO_2_ [31], Ni(OH)_2_ [32], CoO [33]) and conductive polymers (polythiophene [34], polyaniline (PANI) [35], polypyrrole [36]). Among them, polyaniline (PANI) stands out as a conductive polymer with extremely high redox activity [37, 38, 39, 40, 41, 42]. Conventional strategies utilize graphene oxide (GO) as a soft template to enable the in situ growth and anchoring of these pseudocapacitive materials, aiming to synergistically combine electric double‐layer capacitance with faradaic charge storage mechanisms [43]. Though these hybrid materials offer high theoretical capacitance, their practical application is often limited by poor electrical conductivity, structural instability, and sluggish ion diffusion kinetics, which derive from the poorly defined and defective interfaces, preventing systematic optimization of the overall supercapacitor performance. To overcome these limitations, innovative architectural designs are essential. Such designs must achieve the precise synthesis of hybrid structures that combine conductive graphene networks with pseudocapacitive materials [44, 45], without compromising the essential requirements of mechanical stability and rapid ion kinetics [46].

2D laminated hybrid architectures represent a paradigm shift for addressing the aforementioned challenges [46, 47, 48, 49, 50, 51, 52]. Herein, we demonstrate a scalable liquid‐phase assembly strategy for constructing graphene‐based hybrid architecture with nanoscale control over the heterointerface. Defect‐minimized EG is employed as the EDLC component, leveraging its preserved electronic properties and solution processability. Meanwhile, bottom‐up synthesized mesoporous PANI nanosheets function as a structurally engineered, redox‐active pseudocapacitive material. A high‐shear mixing process induces the high‐efficiency and controllable co‐assembly of EG and 2D PANI nanosheets. These components function as mutual surfactants, thereby promoting their face‐to‐face stacking into a well‐defined 2D laminated hybrid architecture. The resulting hybrid material achieves exceptional electrochemical performance, including a high volumetric capacitance of 424.58 F/cm^3^ (1 mV/s) and outstanding rate capability (73.61% retention from 0.2 to 5.0 mA/cm^2^, 54.56% retention from 1–100 mV/s). Such high‐performance originates from the synergistic combination of continuous electron transport via the graphene network, facile ion kinetics through the designed mesoporous PANI structure, and robust heterointerfaces that sustain reversible faradaic reactions. These structure‐property relationships are clearly demonstrated through a series of control experiments, especially by the synthesis of 2D PANI nanosheets without mesoporous structures. Our advanced architecture might establish a new paradigm for rational design of high‐performance energy storage materials.

## 2 Results and Discussion

### 2.1 Construction of 2D EG‐PANI Hybrid Architecture

We adopt the emerging paradigm of 2D hybrid architecture as a novel design strategy for fabricating high‐performance supercapacitor electrodes. The electrochemical exfoliation and characterization of graphene are provided in Figures S1–S5. Defect‐minimized EG serves as the EDLC component, capitalizing on its high electrical conductivity and solution processability. In parallel, we integrate bottom‐up synthesized mesoporous PANI nanosheets, which act as a structurally well‐defined, redox‐active pseudocapacitive material.

**FIGURE 1:**
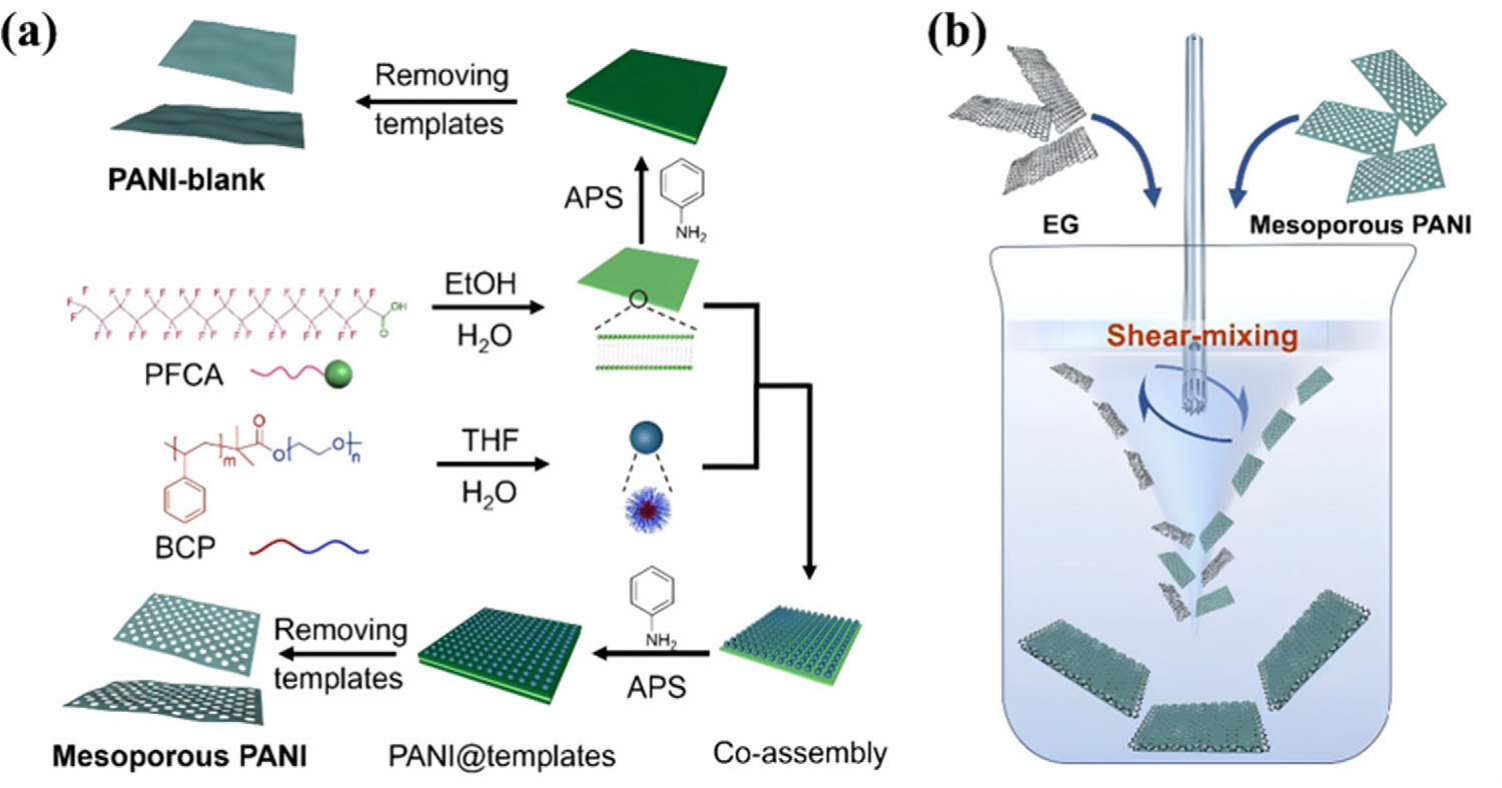
(a) Synthesis protocol of 2D mesoporous PANI nanosheets and 2D PANI‐blank nanosheets. (b) Schematics of the construction of 2D EG‐PANI hybrid architecture via shear‐mixing approach.

The bottom‐up synthesis of 2D PANI nanosheets via soft template approach is illustrated in Figure 1a [36]. 2D mesoporous PANI nanosheets and 2D PANI‐blank nanosheets (without mesoporous structures, as a control experiment) are synthesized accordingly. First, PFCA and BCP are assembled into 2D lipid bilayer structures and spherical micelles, respectively. Upon directly mixing these preformed self‐assemblies, the spherical micelles co‐assemble with the independently existing lipid bilayers via monolayer close‐packing at both interfaces, forming a more complex hierarchical architecture. These structures simultaneously act as templates, thereby confining the growth direction of the polyaniline nanosheets. As a result, mesoscopically ordered porous nanosheets are ultimately obtained. The major difference lies in the employment of BCP in the synthesis procedures. The obtained PANI nanosheets are sonicated in ethanol for 30 min to obtain a dispersion with a concentration of 3 mg/mL, yet partial aggregations can be observed (Figure S6). Subsequently, a predetermined volume of an EG dispersion in ethanol (0.1 mg/mL) is added to the PANI suspension to fabricate hybrid composites with targeted EG: PANI mass ratios of 5:1, 3:1, 1:1, 1:3, and 1:5. The mixed EG and PANI dispersion is then subjected to high‐shear mixing, with the shear rate and processing duration varied systematically, as schematically illustrated in Figure 1b. This high shear‐induced assembly promotes the formation of a well‐ordered 2D hybrid architecture. The process is facilitated by the mutual surfactant behavior of the EG and PANI nanosheets, wherein their 2D morphology enables this co‐stabilization [53]. Furthermore, the graphitic surface of the EG nanosheets promotes the dispersion and alignment of the PANI nanosheets via robust *π–π* interactions. The digital picture of ethanol dispersions of EG, PANI, and EG‐PANI is illustrated in Figure S6.

**FIGURE 2:**
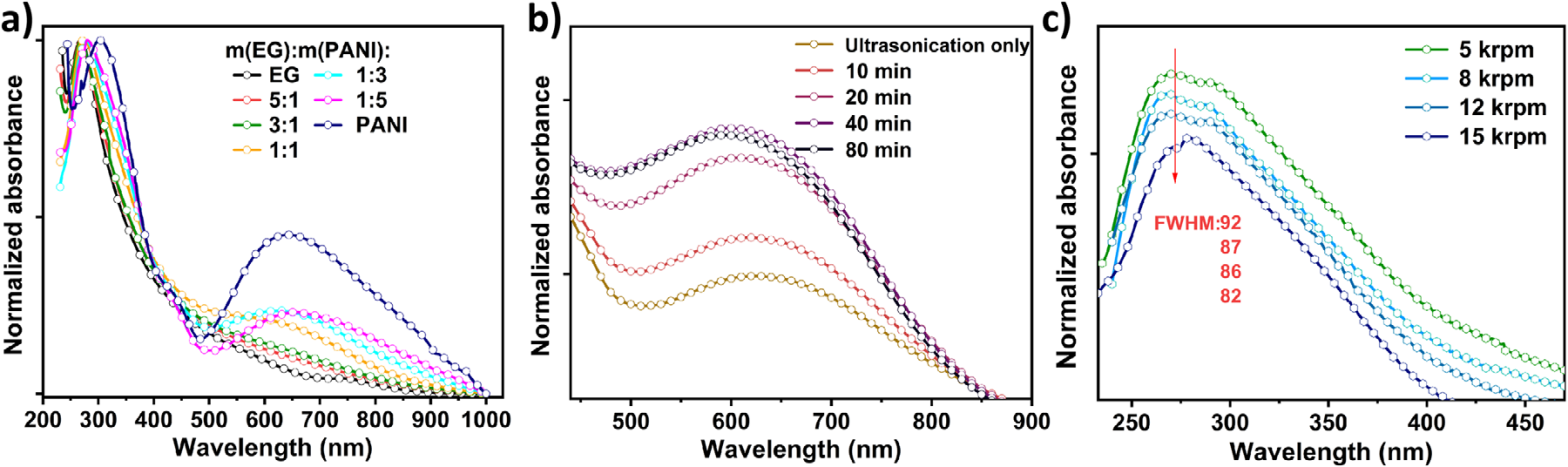
UV–Vis Absorption Spectrum of Ethanol Dispersion of EG‐PANI. (a) The spectra of hybrid architecture with different PANI contents. The spectra of hybrid architecture constructed by different shear‐mixing (b) duration, and (c) rate.

To explore the colloidal properties and electronic interactions within the dispersions, UV–vis absorption spectroscopy analysis is performed (Figure 2). The spectrum of pure EG exhibits a characteristic peak at 275 nm, attributed to the *π–π* ^*^ transitions of the aromatic C─C bonds. With increasing PANI content, this peak undergoes a slight redshift accompanied by weak spectral splitting, indicative of electronic coupling between the EG and PANI nanosheets facilitated by *π–π* stacking. Concurrently, a new absorption feature emerges at approximately 650 nm. This band is ascribed to a charge‐transfer interaction, specifically a *π–π* ^*^ transition between the protonated emeraldine salt form of PANI and the graphitic surface of the EG nanosheets, providing further evidence for the formation of a ground‐state complex in the 2D hybrid architecture. Notably, the characteristic charge‐transfer peak at 275 nm is most sharply defined at a PANI content of 75 wt.% (Figure 2a), indicating the high degree of structural order and colloidal stability for the hybrid architecture in ethanol dispersion. We attribute this optimum to a mass ratio that maximizes the effective *π–π* stacking interface between EG and PANI nanosheets. This synergistic balance enhances the mutual surfactant effect, yielding an exceptionally homogeneous and stable dispersion. These results clearly demonstrate that the principal advantage of our 2D hybrid architecture model over conventional in situ polymerization approaches is the precise control it affords over both the thickness and the mesoporous structure of the pseudocapacitive nanosheets. Specifically, by simply varying the mass ratio of the individual 2D components, an optimized polymer loading can be precisely incorporated into the 2D hybrid architecture while maintaining its predefined nanoarchitecture, including controlled morphology and mesoporosity. Furthermore, this modular 2D hybrid architecture model provides a well‐quantified platform to deconvolute and investigate synergistic effects, enabling a clear assessment of the individual contributions from the graphene and polymer nanosheets.

The effects of shear rate and processing duration on the mixed EG and PANI dispersion are systematically investigated (Figure 2b,c). In contrast to the conventional ultrasonic treatment, the intensity of the *π–π* charge‐transfer absorption band increases progressively with extended shear‐mixing time. This trend indicates that prolonged shear‐mixing treatment facilitates better dispersion of the two components, derived from more extensive and intimate interfacial contact between the nanosheets, thus promoting 2D hybrid architecture formation. A duration of 40 min appears sufficient to drive the assembly toward a stable interface. Furthermore, increasing the shear rate results in a sharpening of the characteristic EG absorption peak. We attribute this to the generation of enhanced hydrodynamic forces and local turbulence within the thin liquid layers, which more effectively overcome the energy barriers to achieve a highly ordered, face‐to‐face arrangement of the nanosheets. Based on these optimization studies, all hybrid architectures are fabricated using a shear rate of 15 000 rpm for a duration of 40 min to ensure complete and uniform assembly.

**FIGURE 3:**
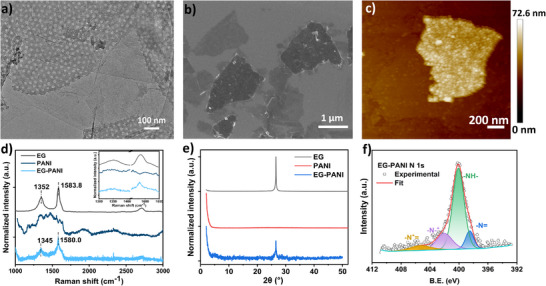
Morphology and spectroscopy characterizations of the obtained 2D EG‐PANI hybrid architecture. (a) TEM (b) SEM and (c) AFM image of the 2D EG‐PANI hybrid architecture deposited on a Si substrate. (d) Raman spectra (performed with a 532 nm laser) of 2D EG‐PANI hybrid architecture. (e) XRD result of 2D EG‐PANI hybrid architecture. (f) High‐resolution N 1s XPS spectrum of 2D EG‐PANI hybrid architecture.

To demonstrate the successful formation of 2D hybrid architecture based on EG and PANI nanosheets, we conduct comprehensive morphological characterization using TEM, SEM, and AFM (Figure 3a–c; Figures S7–S9). Micrographs of the individual components (Figure S7) reveal the distinct mesoporous morphology of the PANI nanosheets. Figure 3a–c depicts the clear overlap between EG and PANI nanosheets. AFM topographical analysis confirms the intimate integration of the two materials, showing PANI nanosheets conformally coated atop EG nanosheets with a uniform thickness of approximately 22 nm (Figure 3c; Figure S7e). This well‐defined layered architecture represents a unique model that effectively prevents the reaggregation of EG nanosheets. Simultaneously, it establishes numerous hierarchical channels for rapid ion transport through the mesopores of PANI and efficient electron transport along the continuous graphene network, thereby addressing key limitations in conventional supercapacitor electrodes.

The Raman spectrum of the 2D hybrid architecture exhibits characteristic vibrational modes of both EG and PANI, confirming the hybrid nature of the material (Figure 3d). The PANI component is identified by its signature peaks: 1164 cm^−1^ (C─H bending of quinoid/benzenoid rings), 1217 cm^−1^ (C─N stretching), 1485 cm^−1^ (C═N stretching of the quinoid ring), and 1576 cm^−1^ (C─C stretching). Crucially, the G‐band of the EG component exhibits a redshift of 3.8 cm^−1^, providing direct evidence of charge‐transfer at the interfaces of the 2D hybrid architecture between the two nanosheets. We attribute this shift to an n‐type doping effect imposed on graphene by the protonated form of PANI. The energy level of the highest occupied molecular orbital (HOMO) in PANI facilitates the transfer of electrons to EG, thereby increasing the electron carrier concentration within the graphene network. This electronic interaction underscores the strong coupling achieved in our structure design. The PXRD pattern of the 2D EG‐PANI hybrid architecture is presented in Figure 3e. The incorporation of PANI nanosheets results in a slight shift of the characteristic graphene (002) diffraction peak to 26.46°. This shift corresponds to an increase in the average interlayer d‐spacing, which can be attributed to the effective separation of graphene layers by the intercalated PANI nanosheets. This intervention weakens the interlayer van der Waals forces that drive reaggregation, thereby promoting a more open and accessible nanostructure.

The chemical states and interfacial interactions within the 2D hybrid architecture are further probed by X‐ray photoelectron spectroscopy (XPS). The high‐resolution N 1s spectrum of the EG‐PANI (Figure 3f) deconvolutes into four characteristic components of PANI: the benzenoid amine at 400.1 eV, quinoid amine at 398.5 eV, nitrogen cationic radical (─N^+^─) at 402.0 eV, and the positively charged nitronium ions ─N^+^ = at 405.3 eV. Meanwhile, the high‐resolution C 1s spectrum (Figure S10a) reveals a binding energy shift of approximately 0.3 eV for the carboxylate (─COO─) peak, from 287.64 to 287.92 eV. This shift provides direct evidence of electronic interaction at the heterointerface, likely arising from charge‐transfer between the functional groups on EG and the nitrogen species in PANI.

### 2.2 Electrochemical Property of 2D EG‐PANI Hybrid Architecture

The electrochemical properties of the 2D EG‐PANI hybrid architecture are assessed using a standard three‐electrode configuration in a 0.5 m H_2_SO_4_ aqueous electrolyte. We construct a hybrid architecture with PANI mass loadings of 25%, 50%, 75%, and 90% for this study. Thin‐film electrodes with a uniform thickness of approximately 25 µm are fabricated on gold‐coated glass substrates via a dry‐transfer method. These samples are denoted as EG‐PANI‐1, EG‐PANI‐2, EG‐PANI‐3, and EG‐PANI‐4, respectively, corresponding to their increasing PANI content. Cyclic voltammetry (CV) and galvanostatic charge–discharge (GCD) measurements are performed to determine the optimal PANI content for maximizing the electrochemical property of the 2D hybrid architecture. As shown in Figure S11, the characteristic redox peaks in the CV curves become progressively more pronounced with increasing PANI content, indicating enhanced faradaic activity and a greater contribution from pseudocapacitance.

The maximum specific capacitance, as indicated by the largest integrated CV area, occurs at a PANI content of 75%. At this optimal loading, the PANI nanosheets are effectively dispersed and electrically wired by the conductive EG network. This architecture ensures optimal electrolyte contact and a highly accessible electroactive surface area, enabling rapid faradaic processes. Conversely, a further increase in PANI content to 90% leads to a noticeable decrease in specific capacitance. We attribute this decline to the incomplete conductive network and the over‐accumulation and aggregation of PANI nanosheets on the graphene surface. This aggregation diminishes the effective surface area available for ion exchange, impedes ion diffusion pathways, and ultimately reduces the overall electrical conductivity of the composite electrode, thereby compromising its electrochemical performance.

However, ion diffusion kinetics become significantly constrained in thicker films, limiting electrolyte access to the entire electroactive surface. To mitigate this mass transport limitation, we fabricate a thinner EG‐PANI‐3 electrode with a controlled thickness of approximately 2–5 µm. With increasing film thickness, the hybrid architecture displays a declined volumetric capacitance. It can be explained by the reduced slopes at low frequency of EIS with increasing film thickness (Figure S12), indicating the difficult ionic diffusion in the thicker film. This reduction in the diffusion pathway length was anticipated to improve ion accessibility throughout the electrode bulk, thereby boosting the specific capacitance.

**FIGURE 4:**
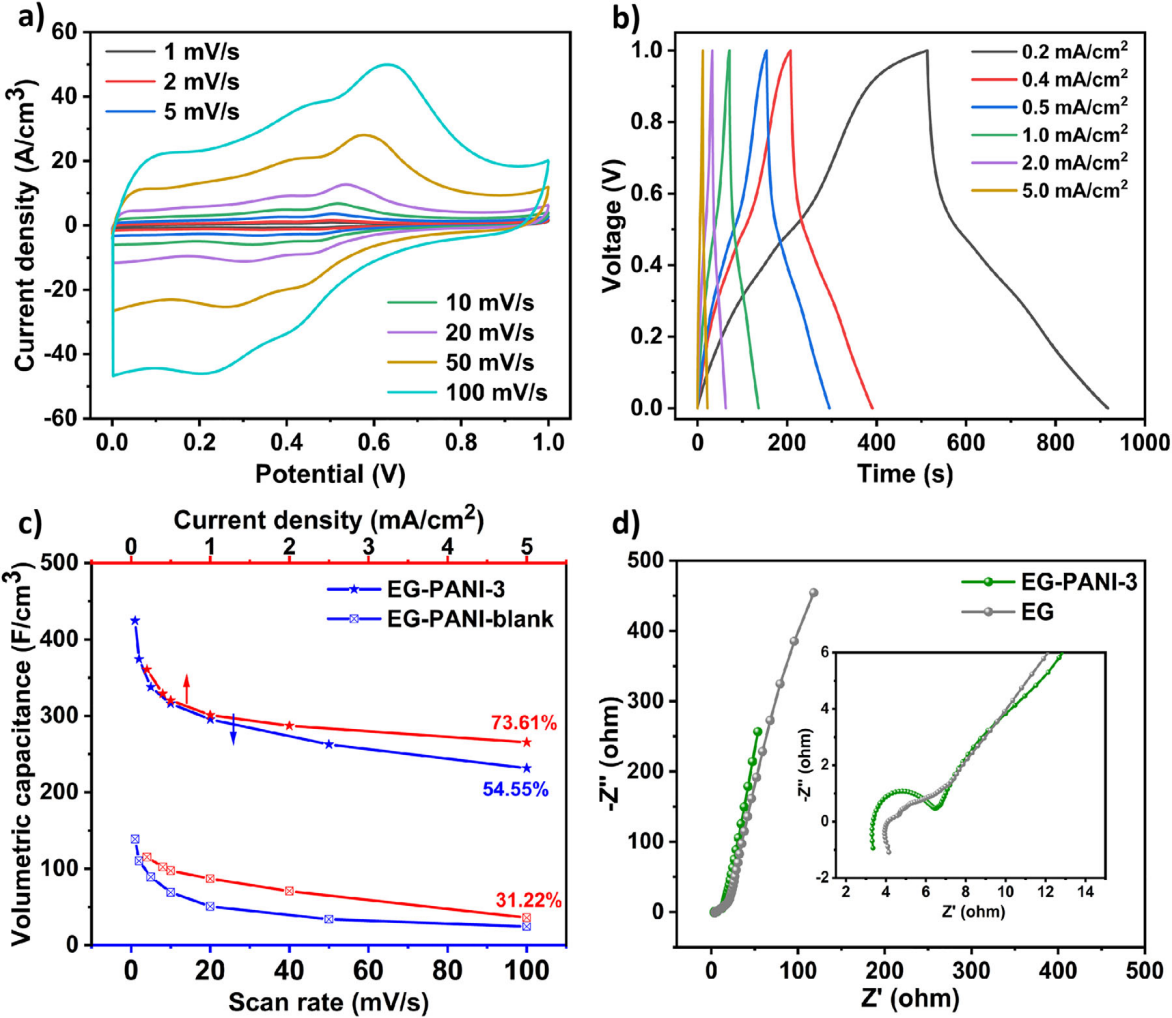
Electrochemical characterization of EG‐PANI‐3. (a) CVs of EG‐PANI‐3 obtained at different scan rates of 1–100 mV/s. (b) GCDs of EG‐PANI‐3 tested at current densities of 0.2–5.0 mA/cm^2^. (c) Volumetric capacitance of EG‐PANI‐3 and EG‐PANI‐blank. (d) EIS of EG and EG‐PANI‐3.

Figure 4a presents the cyclic voltammetry (CV) profiles of the optimized EG‐PANI‐3 film in 0.5 m H_2_SO_4_, recorded at scan rates from 1 to 100 mV/s. The CV curves retain a quasi‐rectangular shape even at elevated scan rates, confirming the highly reversible nature of the charge storage process and the synergistic coexistence of EDLC and faradaic pseudocapacitance. All CV curves exhibit two distinct pairs of redox peaks, which are characteristic of PANI's faradaic transitions. The first pair is attributed to the transformation of the fully reduced state to emeraldine (semi‐oxidized), while the second pair corresponds to the subsequent conversion of emeraldine to the fully oxidized state. The GCD profiles of the EG‐PANI‐3 electrode, measured within a 0–1 V potential window, are presented in Figure 4b. The curves deviate from the ideal linear profile of an electric double‐layer capacitor, exhibiting distinct curvature and plateaus. This non‐ideal behavior provides further confirmation of the dominant pseudocapacitive contribution from the PANI component, a conclusion that is fully consistent with the redox peaks observed in the CV data.

The specific volumetric capacitance of the EG‐PANI‐3 electrode is calculated from both CV and GCD results, yielding outstanding values of 424.58 and 360.76 F/cm^3^ at a scan rate of 1 mV/s and a current density of 0.2 mA/cm^2^, respectively. To deconvolute the individual contributions, the volumetric capacitances of the pure EG and PANI are separately measured under identical conditions (Figures S13 and S14). Remarkably, the capacitance of the EG‐PANI‐3 significantly exceeds the arithmetic sum of the contributions from its individual constituents, calculated based on their mass fractions. This clear positive deviation provides direct and quantitative evidence of a strong synergistic effect between the conductive EG network and the faradaically active PANI, enhancing the performance beyond what either material could achieve independently.

The EG‐PANI‐3 electrode demonstrates exceptional rate capability, which is a critical metric for a high‐power supercapacitor. The CV and GCD curves retain their characteristic shapes with increasing scan rate and current density, indicating highly reversible kinetics. Notably, as illustrated in Figure 4c, even at a high scan rate of 100 mV/s (increased by 100 times) and a current density of 5.0 mA/cm^2^, the electrode maintains impressive volumetric capacitances of 231.62 and 265.47 F/cm^3^, respectively. This corresponds to an outstanding capacitance retention of 73.61% relative to its low‐rate performance. We attribute this combined high volumetric capacitance and superior rate capability to the synergistic advantages of our designed architecture. The alternating, laminated hybrid architecture effectively prevents the reaggregation of both EG and PANI nanosheets. This maximizes the utilization of the graphene's high specific surface area and intrinsic conductivity, significantly increasing the electroactive interface with the electrolyte to enhance overall charge storage and facilitate efficient electron transport. Concurrently, this layered and mesoporous nanoarchitecture creates continuous, short pathways for rapid ion diffusion throughout the electrode bulk, minimizing transport resistance for both electrons and electrolyte ions.

To elucidate the critical role of the engineered mesoporosity within the PANI nanosheets, a control sample is prepared using PANI nanosheets without mesoporous structure (denoted as PANI‐blank) (morphologies shown in Figure S15), and the resulting 2D hybrid architecture is denoted as EG‐PANI‐blank (Figure S16). The EG‐PANI‐blank control sample, which lacks this engineered mesoporosity, severely hinders ion transport. At high current densities, the inability of electrolyte ions to readily access the internal surface area results in a drastic drop of rate capability, as evidenced by its significantly lower capacitance retention of only 31.22% (Figure 4c; Figure S17).

To elucidate the electrochemical reaction kinetics, we perform electrochemical impedance spectroscopy (EIS) on the EG‐PANI‐3 electrode (Figure 4d). The Nyquist plot for EG‐PANI‐3 shows a smaller real‐axis intercept at high frequency as compared to pure EG, indicating a lower equivalent series resistance (R_s_). The diameter of the semicircle in the high‐frequency region corresponds to the charge‐transfer resistance (R_ct_). The R_ct_ for EG‐PANI‐3 with a value of 2.52 Ω is moderately higher than that of pure EG. This is expected, as the introduction of the semiconducting PANI component introduces additional interfacial charge‐transfer barriers that are not present in the highly conductive, pure graphene network. Despite this, in the low‐frequency region, EG‐PANI‐3 exhibits a much steeper Warburg diffusion tail that approaches a vertical line. This indicates exceptionally facile ion diffusion within the mesoporous architecture and near‐ideal capacitive behavior, which overwhelmingly compensates for the modest increase in R_ct_. The synergistic combination of graphene's conductivity and PANI's mesoporosity ultimately results in superior overall electrochemical kinetics for the hybrid architecture.

To gain deeper insights into the kinetic processes of the EG‐PANI composite electrodes, distribution of relaxation times (DRT) analysis was performed on the electrochemical impedance spectra of different electrode configurations (Figure S18). The EG exhibits a distinct high‐frequency relaxation peak at τ ≈ 0.03 ms, which is attributed to the charge‐transfer process at the electrolyte/electrode interface. After the formation of the EG‐PANI composite structure, this peak shifts significantly leftward to τ ≈ 0.017 ms, indicating a shortened relaxation time for the charge‐transfer process. However, the peak intensity increases slightly, suggesting a modest increase in charge‐transfer resistance due to the introduction of the PANI layer at the interface. Notably, the diffusion peaks in the low‐frequency region (τ ≈ 0.05 and 0.29 s) also shift noticeably leftward compared to EG and EG‐PANI‐blank, confirming that the short‐range diffusion channels provided by the 2D mesoporous PANI nanosheets, in synergy with the graphene conductive network, optimize ion transport kinetics. This result directly reveals the structural origin of the excellent rate capability in this work: the shear‐induced laminated stacked structure not only forms stable interfacial contact but also constructs efficient ion diffusion pathways through the 2D mesoporous PANI nanosheets, corroborating the results from rate capability tests.

To further investigate the cycling stability of the EG‐PANI‐3 electrode, we performed 2400 CV cycles on EG‐PANI‐3 at a scan rate of 0.1 V/s, and the capacitance retention was 90.5%. (Figure S19). Raman spectroscopy was performed on the electrode before and after 2400 cyclic voltammetry tests (Figure S20). The results show that the G‐band (1580 cm^−1^) of graphene maintained a significant redshifted state after cycling. This indicates that the *π–π* interfacial interaction between EG and PANI was well maintained after long‐term cycling, with no large‐scale interfacial delamination or failure. This stable interfacial structure provides the structural basis for sustained charge‐transport pathways. Notably, the I_D_/I_G_ of graphene increased slightly. This modest increase may be attributed to minor perturbations of the graphene surface caused by PANI chain relaxation during long‐term redox processes, or to a small number of structural defects introduced during repeated doping/dedoping processes. Nevertheless, this I_D_/I_G_ remains lower than that of reduced graphene oxide (rGO), confirming that the intrinsic structural integrity of EG was well maintained after cycling. These results indicate that the considerable cycling stability of the EG‐PANI electrode originates from (i) the stable *π–π* interaction, ensuring sustained charge‐transfer pathways; (ii) the buffering effect of the 2D mesoporous structure against volume collapse. Although the interface and framework structure remained generally stable, the electrochemical degradation of PANI during long‐term cycling remains an important direction for future optimization.

**FIGURE 5:**
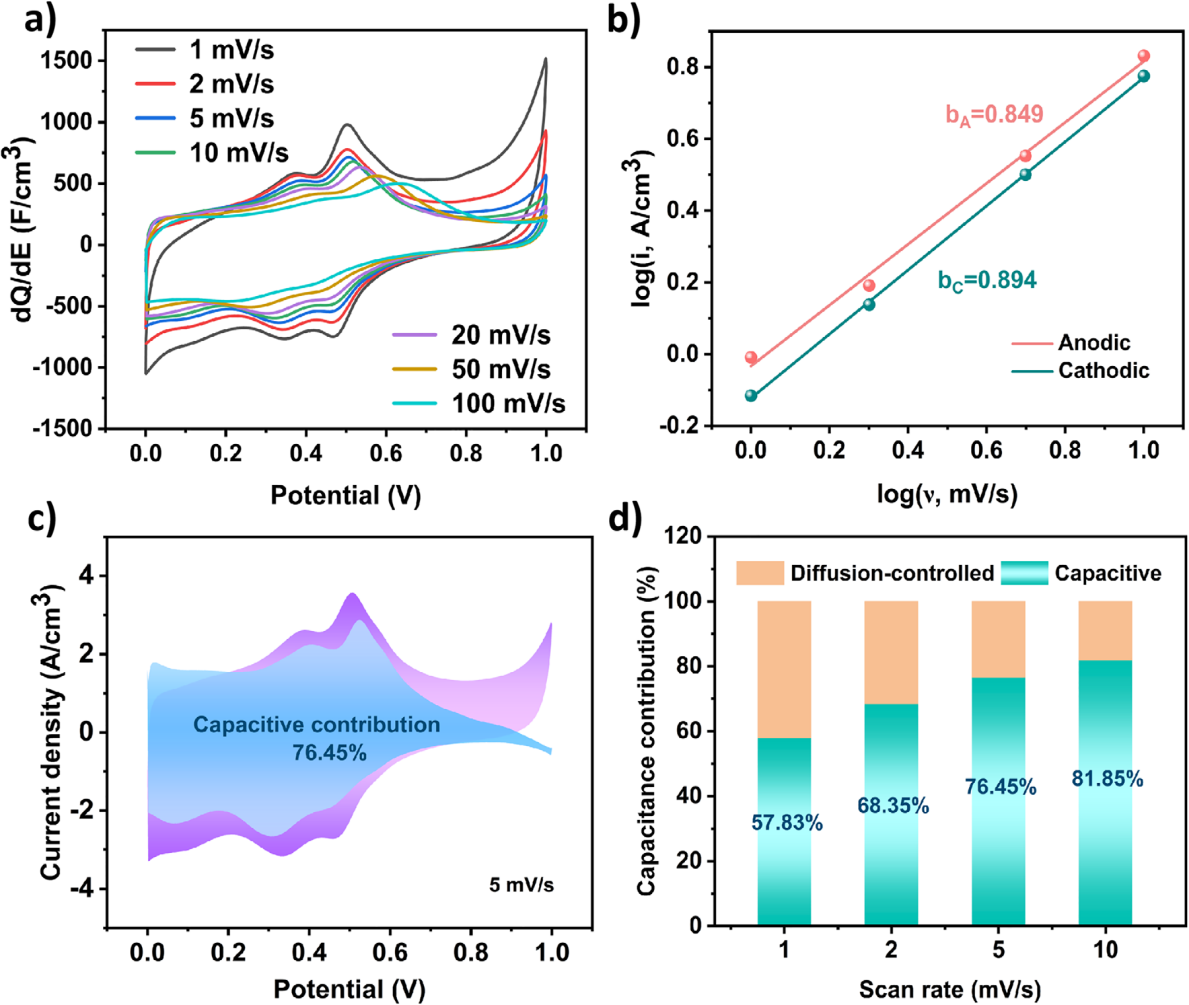
Calculation of Charge Storage Dynamics for EG‐PANI‐3. (a) Differential capacitance versus potential curves at different scan rates. (b) Linear fit of log(*i*) versus log(ν) for anodic peak and cathodic peak. (c) capacitive and diffusion‐controlled contribution to charge storage at 5 mV/s. (d) percentages of capacitive and diffusion‐controlled capacities at different scan rates of EG‐PANI‐3 in 0.5 m H_2_SO_4_.

The charge storage dynamics of the EG‐PANI‐3 electrode are further investigated by analyzing the scan‐rate dependence of the CV profiles. As shown in Figure 5a, the differential capacitance gradually decreases with increasing scan rate, indicating that the redox processes, while largely surface‐controlled, exhibit a non‐negligible diffusion‐limited component. To quantitatively deconvolute the storage mechanisms, the b‐value is calculated from the power‐law relationship between peak current (i) and scan rate (ν):

(1) $$i=a\nu^{b}$$

The b‐values for both the anodic and cathodic peaks are found to be close to 1.0 (Figure 5b), confirming that the charge storage is predominantly governed by a rapid, surface‐controlled capacitive process. Subsequently, the quantitative contribution ratio of the capacitive versus diffusion‐controlled processes is calculated using the following equation:

(2) $$i=k_{1}\nu +k_{2}\nu^{1/2}$$

As shown in Figure 5c, the capacitive contribution accounts for 76.45% of the total charge storage at a scan rate of 5 mV/s. Remarkably, this contribution increases progressively with scan rate, reaching 81.85% at 10 mV/s. This trend, as summarized in Figure 5d, demonstrates that capacitive processes overwhelmingly dominate the energy storage mechanism, particularly at high operating rates. The superior rate capability of the EG‐PANI‐3 can be directly attributed to this high and increasing fraction of surface‐controlled kinetics, which are hardly limited by solid‐state diffusion. Therefore, the high capacitance of the EG‐PANI‐3 originates from a synergistic combination of two primary mechanisms: (1) robust faradaic pseudocapacitance from the redox reactions of the quinoid/benzenoid nitrogen species (e.g., C─N^+^) within the PANI framework, and (2) EDLC enabled by the high specific surface area and well‐defined mesoporosity of the integrated nanostructure.

### 2.3 Electrochemical Performance of Supercapacitor Based on EG‐PANI

**FIGURE 6:**
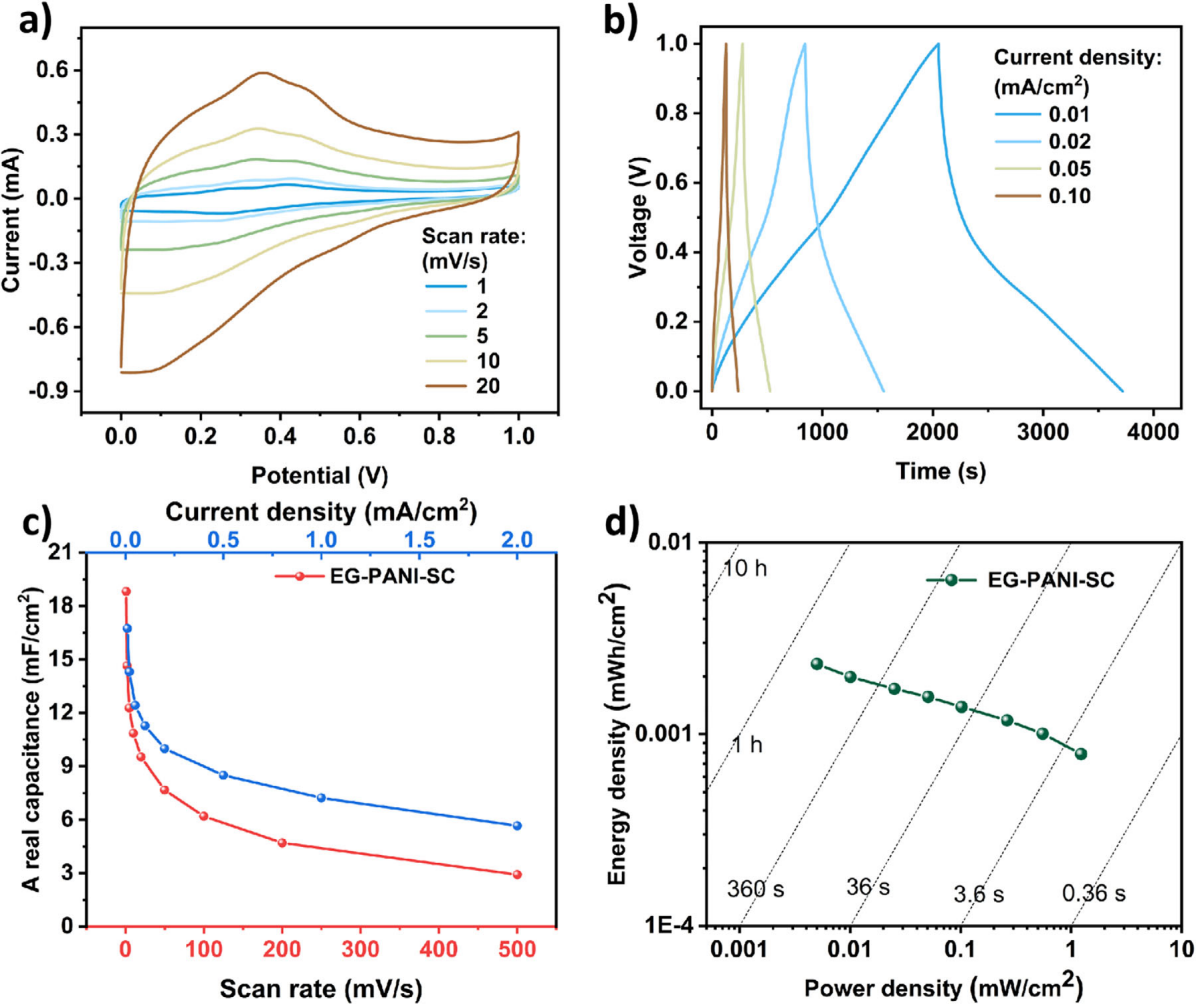
(a) CV curves (scan rates ranging from 1 to 20 mV/s), (b) GCD curves (charging/discharging current ranging from 0.01 to 0.10 mA/cm^2^), (c) Areal capacitance at different current density and scan rate, (d) Ragone plot of EG‐PANI‐SC.

To further investigate the performance of EG‐PANI‐3 as an electrode material in practical energy storage applications, planar patterned interdigitated electrodes based on EG‐PANI‐3 were fabricated by screen printing onto a polyimide film substrate with pre‐evaporated gold as the current collector. Subsequently, a gel H_2_SO_4_/PVA electrolyte was drop‐cast and allowed to solidify overnight to prepare planar supercapacitors (SCs). A schematic illustration and detailed specifications are shown in Figure S21. Its electrochemical behavior was first evaluated through cyclic voltammetry. The SC exhibited a series of near‐rectangular CV curves at different scan rates (Figure 6a; Figure S22a), operating reliably up to a scan rate of 500 mV/s, indicating its stable capacitive performance. Based on the CV curves, the areal capacitances of the SC were calculated to be 18.82, 7.67, and 2.92 mF/cm^2^ at scan rates of 1, 50, and 500 mV/s, respectively. The GCD curves with near‐triangular shapes further confirmed the excellent capacitive performance of the SC (Figure 6b; Figure S22b). Additionally, the device still demonstrated remarkable rate capability, with a capacitance retention rate of 33.9% as the current density increased from 0.01 to 2.0 mA/cm^2^ (The areal capacitance decreased from 16.74 to 5.67 mF/cm^2^) (Figure 6c). The overall performance of the SC was studied using a Ragone plot (Figure 6d), which displays the areal energy density and power density of the entire device. The SC provided energy densities of 0.0023, 0.0014, and 0.00079 mWh/cm^2^ at power densities of 0.005, 0.102, and 1.231 mW/cm^2^, respectively. As shown in Figure S23, two SCs connected in series can power a small LED bulb. To evaluate the practical cycling stability of the EG‐PANI, the supercapacitor device was subjected to CV cycling for 5000 cycles. As shown in Figure S24a, the device retained 76.4% of its initial specific capacitance after 5000 cycles, with the CV curve shape remaining essentially unchanged. The capacitance decay predominantly occurred during the first 1000 cycles (≈17% loss), after which the degradation gradually slowed and entered a relatively stable plateau. This cycling performance reveals the inherent challenges associated with PANI‐based electrodes. The initial capacity loss is primarily attributed to: Intrinsic volume changes of PANI during repeated redox reactions, which induce a gradual detachment of some active domains from the conductive network; Partial dedoping or irreversible oxidation of PANI chains in acidic electrolyte. EIS before and after cycling tests are shown in Figure S24b. After 5000 cycles, there is a decrease in the slope of the straight line and an increase in resistance, indicating structural changes within the electrode material. The ion diffusion is hindered. Additionally, the SC exhibited outstanding mechanical flexibility, with excellent electrochemical stability confirmed by the consistent CV curves regardless of bending deformation (Figure S25).

## 3 Conclusion

We demonstrate a scalable strategy for constructing high‐performance supercapacitor electrode materials through interfacial assembly of electrochemically exfoliated graphene and mesoporous polyaniline into a 2D hybrid architecture by shear‐mixing. This design simultaneously overcomes the key limitations of conventional composites by combining defect‐minimized conductive pathways, engineered ion‐diffusion channels, and stable faradaic interfaces, achieving high volumetric capacitance with outstanding rate capability. Our approach establishes a new paradigm for scalable production of advanced energy storage materials that bridges critical gaps in power and energy density. Although the 2D mesoporous structure and the *π–π* interface synergistically enhance electrochemical performance, further improving the long‐term cycling stability by mitigating PANI activity degradation represents a promising direction for future research. Addressing this common challenge in PANI‐based materials will pave the way for their practical application in high‐performance energy storage devices.

## 4 Experimental Methods

### 4.1 Pretreatment of Graphite Foil

Cut the graphite foil (0.13 mm, Alfa Aesar) into 1 × 3 cm pieces, fully immerse them in liquid nitrogen for 1 min, and then quickly transfer them to anhydrous ethanol. Repeat this process three times, and then air‐dry the pieces at room‐temperature.

### 4.2 Electrochemical Production of EG Nanosheets

Select graphite foil as the anode and a Pt sheet (Alfa Aesar) as the cathode. Immerse both of them in the electrolyte solution (a mixture of 0.1 m NaOH and 0.1 m Na_2_SO_4_ in a volume ratio of 9:1). With a distance of 2 cm between the graphite and Pt sheet, apply a direct current voltage of 10 V to conduct electrochemical exfoliation of the graphite foil. Subsequently, collect the exfoliated products by filtration using a PTFE membrane (pore size 0.22 µm, purchased from Merck Millipore), and wash them repeatedly with deionized water and anhydrous ethanol. Finally, disperse the obtained products in anhydrous ethanol by sonication at a power of 360 w for 1 h, let them stand for more than 24 h, and then collect the supernatant for subsequent characterization.

### 4.3 Preparation of EG‐PANI 2D Laminated Hybrid Architecture

The synthesis method of 2D mesoporous PANI and 2D PANI‐blank was conducted as previously reported [36]. Typically, spherical micellar aggregates were prepared by dissolving 0.050 g of PS_117_‐b‐PEO_114_ block copolymer (BCP) in a mixed solvent of 1 mL THF and 8 mL H_2_O. Following thorough stirring, 0.024 g of perfluorooctadecanoic acid solution (13.3 mg/mL in ethanol) was introduced. Subsequently, aniline, HCl, and ammonium persulfate (APS, 0.076 g) were added at a molar ratio of 5:5:6. Continuous stirring induced a color transition of the solution to dark green, signifying the polymerization of aniline monomers into PANI. The pristine mesoporous PANI nanosheets were finally collected after repeated washing with THF, ethanol, and water to remove BCPs, PFCA, and excess ions. Without using BCP/PFCA templates, PANI‐blank was successfully synthesized. The ethanol dispersions of PANI nanosheets (3 mg/mL) and EG nanosheets (0.1 mg/mL) were mixed at different volume ratios and then sonicated for 30 min. High‐shear forces provided by a shear‐mixer were utilized to mix them in the solvent (more details are in the Supporting Information).

### 4.4 Preparation of Working Electrodes for Three‐Electrode Measurements

This work employs a dry transfer method to prepare electrode films on gold‐evaporated glass substrates, and does not use “theoretical material density” for volumetric capacitance conversion. The preparation process for three‐electrode test samples is as follows: first, gold was evaporated onto a glass slide at a rate of 0.01 nm/s until a thickness of 30 nm was reached. Using a filtration apparatus with specified dimensions, a certain volume of EG‐PANI dispersion was filtered through a PTFE membrane to form a filter cake, with residual solvent retained to keep the filter cake moist. The membrane was then peeled off, and the electrode material filter cake was inverted onto the gold surface, pressed firmly for approximately 2 min until the solvent was extruded and dried, after which the membrane was gently peeled away. The glass slide was placed in an 80°C oven for vacuum annealing for 12 h, then removed for testing.

The film area depends on the filtration apparatus used and the shape of the filter cake formed during filtration, resulting in a circular shape with a diameter of 1.6 cm. When determining the optimal PANI content for EG‐PANI electrochemical performance, thicker films were used, and the effect of film thickness on the electrochemical reaction was briefly investigated. For a detailed investigation of EG‐PANI‐3, thinner films were used, and the data in Figures 4 and 5 were obtained based on films with a thickness of 2 µm. By controlling the concentration of the dispersion and the filtration volume, the film thickness can be controlled within the range of 2–5 µm, although precise control at the 0.1 µm level cannot be achieved. The shape of the film prepared by the dry transfer method was the same as the shape of the filter cake during filtration, which was circular and covered the gold surface.

Thickness measurements were performed using a Bruker Dektak150 surface profilometer with a 12.5 µm stylus. During scanning, the initial scanning position of the stylus was positioned on the bare evaporated gold layer, and scanning was performed linearly along the diameter direction of the film. The electrode exhibits a continuous, dense stacked morphology without obvious local collapse or agglomeration, providing direct structural evidence for thickness uniformity. The average thickness was determined using Dektak software or Origin software. The volumetric was calculated by using the following formula:

V=A×d

### 4.5 Materials Characterization

The zeta potential of the EG nanosheets in water dispersion was tested using a Zetasizer Pro (produced by Malvern Panalytical Ltd.). The morphology was examined using SEM (Regulus 8100, the operating voltage is 3.0 kV), TEM (JEM‐2100F, the operating voltage is 200 kV), and AFM (BRUKER ICON‐XR, the images were obtained under the tapping mode with the probe whose mode is RTESPA‐300). Raman spectra were recorded by a Grating‐based Raman spectrometer with 532 nm laser excitation. The silicon peak at 520.7 cm^−1^ served as a reference for wavenumber calibration. X‐ray diffraction (XRD) spectra were obtained on an Intelligent X‐ray diffractometer (Produced by Rigaku Corporation). XPS analysis was performed by a Thermo Scientific KAlpha X‐ray photoelectron spectrometer, which was equipped with an aluminum X‐ray source (energy 1.4866 keV) at a vacuum level of 10^−8^–10^−9^ mbar in the main chamber. The spot size of the X‐ray beam was fixed at 400 µm. Survey spectra were recorded as an average of 10 scans with a pass energy of 200.00 eV and a step size of 1 eV; high‐resolution spectra were an average of 10 scans with a pass energy of 50.00 eV and a step size of 0.1 eV.

### 4.6 Electrochemical Measurement

The electrochemical performances were performed by an electrochemical workstation (CHI760E), using CV measurements at different scan rates from 1 to 100 mV/s, GCD profiles at different current densities, and EIS recorded in the frequency range from 0.01 Hz to 100 kHz with an ac amplitude of 5 mV.

## Conflicts of Interest

The authors declare no conflicts of interest.

## Supporting information




**Supporting File**: smll73262‐sup‐0001‐SuppMat.docx.

## Data Availability

The data that support the findings of this study are available from the corresponding author upon reasonable request.
