# Supplementary material for: 2D Laminated Hybrid Architectures Based on Graphene/Mesoporous Polyaniline for High‐Performance Supercapacitor Electrode
Source: Small. 2026 Mar 31;22(29):e00004. doi: 10.1002/smll.202600004 (PMC13206345; doi:10.1002/smll.202600004)
Supplement: Supplementary file 1 — Supporting File: smll73262‐sup‐0001‐SuppMat.docx. [file SMLL-22-e00004-s001.docx]

Supporting Information

2D Laminated Hybrid Architectures Based on Graphene/Mesoporous Polyaniline for High-Performance Supercapacitor Electrode

Jiashu Li, Chenchen Zhang, Hongyi Zhang, Liangyu Dong, Bin Han, Leijing Liu, Shaohua Liu,* Zhaoyang Liu,* Wenjing Tian*, and Paolo Samorì*

**Calculation.**

The capacitance values were calculated from the curves of CV according to the following equations (1):

*C_1_=* $\frac{1}{\nu\Delta V}\int I(V)dV$ (1)

Where *ν* is the scan rate, $\Delta V$ is the potential window range of CV curve. *I*(*V*) is the voltammetry current.

Volumetric capacitance were calculated according to the formula (2):

$C_{material}^{volumetric}$=*C_1_* /$V_{electrode}$ (2)

Where *Velectrode* is the volume of the electrode material in a three-electrode system.

The capacitance values were calculated from the discharge curves of GCD according to the following equations (3):

*C_2_* = $\frac{I\Delta t}{\Delta V-IR}$ (3)

where $\Delta t$ refers to the discharge time. *I* is the discharge current. *IR* represents the voltage drop at the beginning of the discharge process, caused by internal resistance of the system.

Volumetric capacitance was calculated according to the formula (4):

$C_{material}^{volumetric}$*=C_2_* /$V_{electrode}$ (4)

**Shear Method.**

Using the T 25 easy clean digital shear-mixer manufactured by IKA, paired with the S 25 EC - C - 25 F Dispersing tool for shear mixing. The tool head consists of a rotor and a stator, with the rotor featuring 6 blades and the stator having 12 narrow slots. The diameters of the rotor and stator are 18 mm and 25 mm, respectively, with a gap of 0.5 mm. It can provide a maximum velocity of 25000 rpm and a maximum circumferential speed of 23.6 m/s. During rotation, the liquid is driven by centrifugal force and subjected to intense shear in the gap and slits between the stator and rotor.

In an experiment for constructing heterostructures, the mixed dispersion is added to a container, and then the dispersing tool head is lowered below the liquid surface. The immersion depth of the tool head is controlled to exceed 35 mm, with the tool head positioned at the center of the container's cross-section. The distance between the lower end of the tool head and the bottom of the container is kept above 1 cm to prevent the formation of asymmetrical vortexes and ensure uniform circulation of the liquid and solids. The velocity is set by adjusting the knob, and the time is set by adjusting the screen buttons. After starting, the shear-mixer gradually increases the velocity to the set value and operates at this speed for the preset time.

Preparation of EG nanosheets

Prior to exfoliation, the graphite foil undergoes a cryogenic pretreatment involving three cycles of alternating immersion in liquid nitrogen (1.5 min) and anhydrous ethanol (0.5 min). This process rapidly weakens interlayer van der Waals forces through instantaneous vaporization of liquid nitrogen and ethanol solidification, collectively yielding a ~10 times volumetric expansion. The pretreated graphite foil is then electrochemically exfoliated in a 0.1 M NaOH/0.1 M Na_2_SO_4_ aqueous electrolyte using a two-electrode configuration (10V, DC), with the expanded graphite as anode and platinum foil as cathode (Figure S1a).

The electrochemical exfoliation process simultaneously generates EG flakes while controlling oxidation through the following key steps: (1) Hydroxyl radicals (•OH) produced by water electrolysis selectively functionalize graphite grain boundaries, (2) oxygen-containing radicals minorly oxidize sp^2^-carbon domains, promoting graphite layer depolarization and interlayer expansion, and (3) intercalation of SO_4_^2-^ anions and electrolytic gases further weakens van der Waals forces between adjacent graphite layers. These synergistic process yields stable EG dispersions (0.5 mg/mL) after repeated washing steps and mild sonication (360 W, 30 min) (Figure S1b). Compared to conventional methods, this approach avoids the extreme oxidation or low yields of traditional techniques, making it highly promising for future industrial applications.^[1, 2]^

Comprehensive microscopic analysis confirms the high quality and monolayer nature of the EG nanosheets (Figure S2-3). Transmission electron microscopy (TEM) imaging reveals transparent and ultrathin flakes with minimal wrinkles (Figure S4a), while the characteristic hexagonal diffraction pattern in selected-area electron diffraction (SAED) confirms a well-preserved crystalline structure with Bernal stacking. Scanning electron microscopy (SEM) characterization demonstrates exceptional morphological uniformity, with more than 80% of flakes maintaining 2-3 μm lateral dimensions and smooth surfaces (Figure S4b). Atomic force microscopy (AFM) topographic analysis measures a characteristic thickness of 0.76 nm (Figure S4c), corresponding to monolayer graphene, with surface roughness of 0.2 nm. Raman spectroscopy reveals the structural quality of the EG, showing characteristic D (1352 cm^-1^), G (1583 cm^-1^), and 2D (2711 cm^-1^) peaks with an ID/IG ratio of 0.57, indicating moderate defects while maintaining good crystallinity (Figure S4d). XPS analysis confirms minimal oxidation (C/O ratio>7), with quite low oxygen-containing groups (carboxyl, hydroxyl) introduced during electrochemical exfoliation (Figure S4f and Figure S5). The colloidal stability of the obtained aqueous dispersion is evidenced by a zeta potential of -38.47 mV (Figure S4e), attributed to these surface functional groups. The powder X-ray diffraction (PXRD) pattern showed a sharp diffraction peak at 26.56° (002) corresponding to an interlayer d-spacing of 3.36 Å (Figure S4g), consistent with high-quality graphene samples. Together with TEM/SEM/AFM results, these analyses confirm the successful production of few-layer EG flakes (1-2 layers) with controlled defect density and excellent dispersibility.

**Supplementary figures and tables**


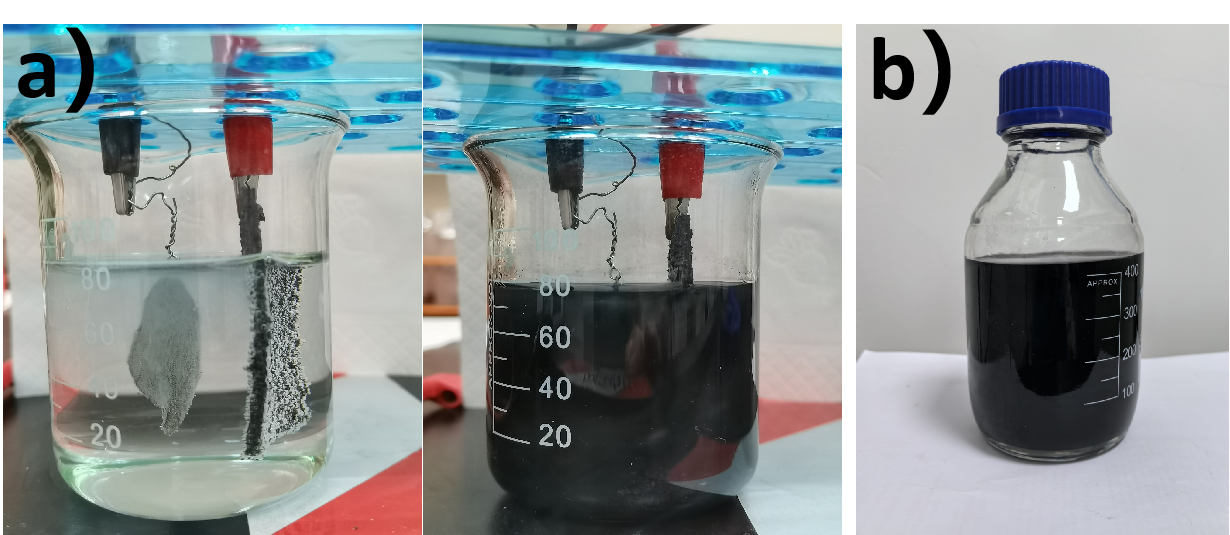


**Figure S1.** (a) photograph of the experimental setup for ongoing and completed electrochemical exfoliation of graphene. (b) Photograph of EG dispersion in ethanol absolute.


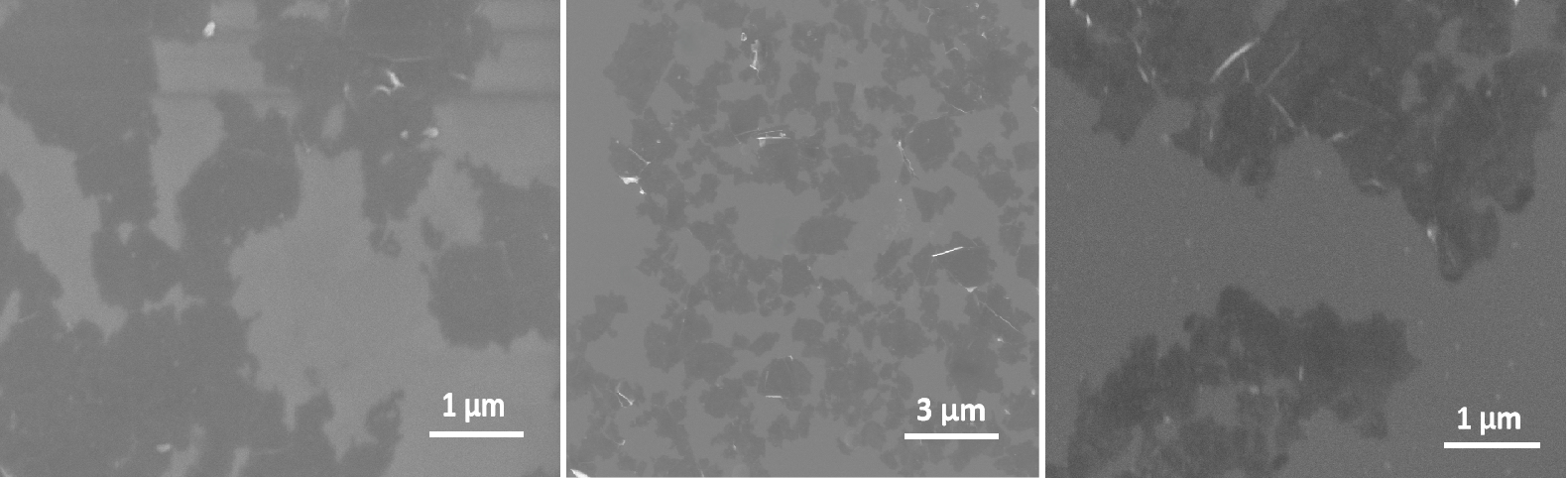


**Figure S2.** SEM images of EG nanosheets.


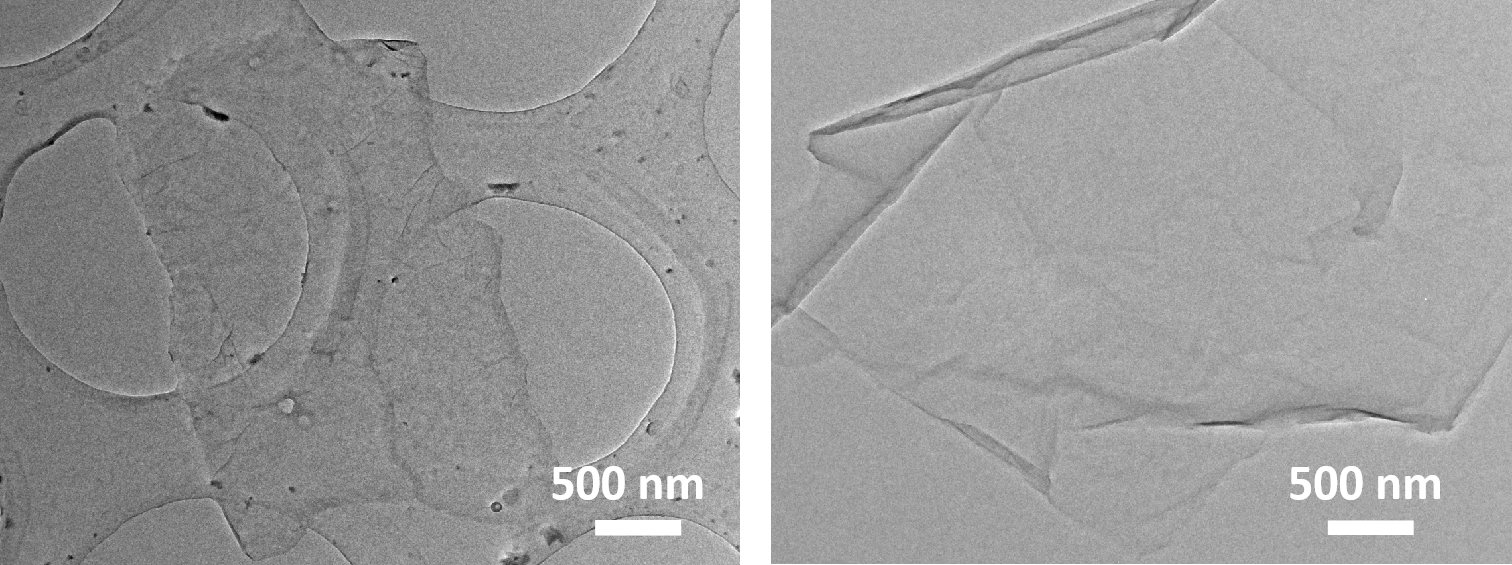


**Figure S3.** TEM images of EG nanosheets.


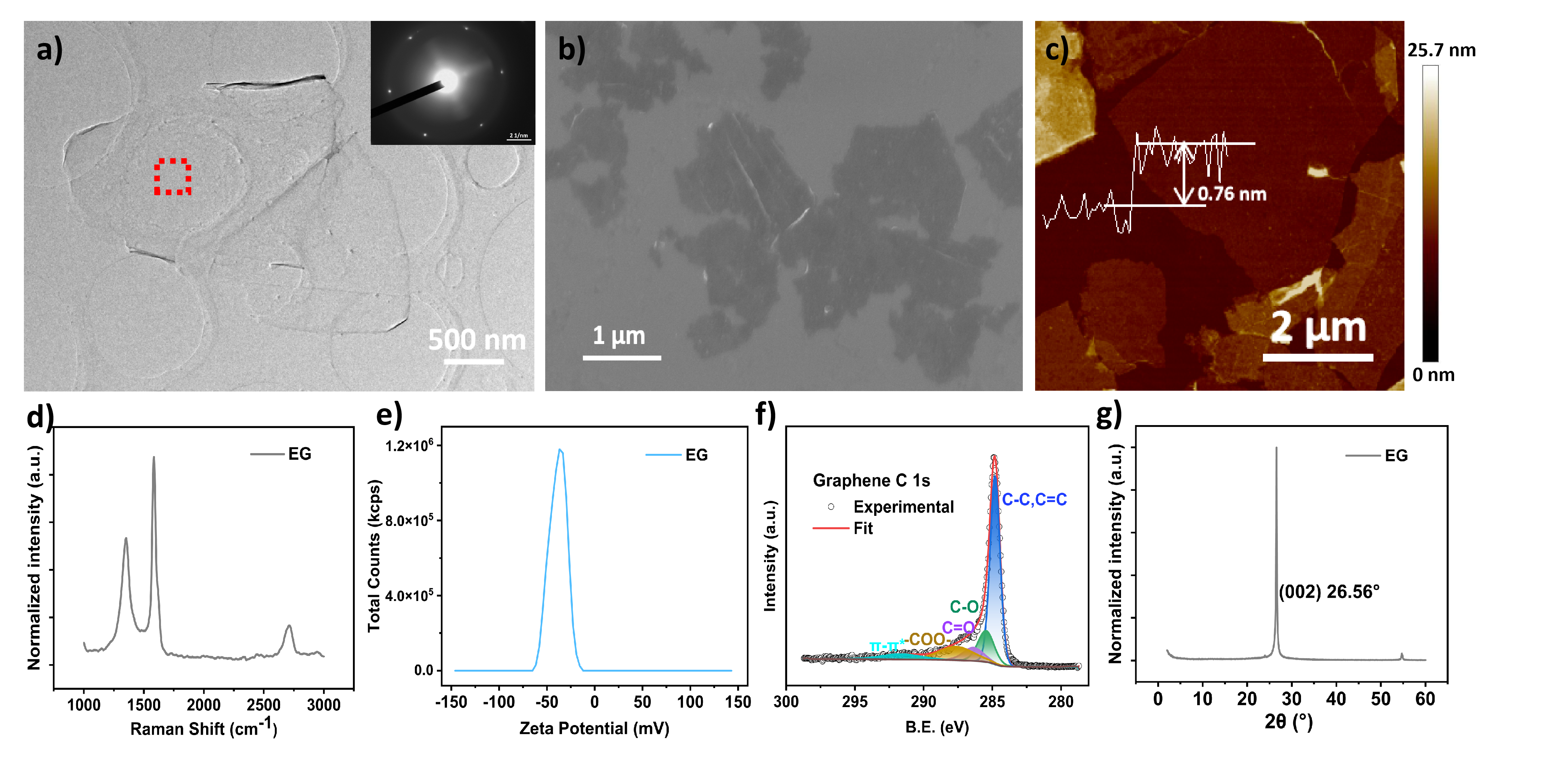


**Figure S4.** Morphological and spectroscopic characterizations of the obtained graphene nanosheets. (a) TEM images of EG nanosheet. The inset is the SAED pattern of the area marked by the red dashed line in EG nanosheet. (b) SEM image of EG nanosheets deposited on a Si substrate. (c) AFM image of EG nanosheets on Si substrate. (d) Raman spectra (excited by 532 nm laser) of EG nanosheet. (e) Z-potential measurement of EG water dispersion. (f) high-resolution C 1s XPS spectrum of EG nanosheets. The five peaks that appear can be attributed to C=C/C-C with binding energy (B.E.) at 284.80 eV, C-O with B.E. at 285.46 eV, C=O with B.E. at 286.38 eV, -COO- with B.E. at 287.64 eV and π-π interaction with B.E. at 291.65 eV. The total oxygen content of EG is only 8.9%. (g) XRD spectra of EG nanosheets.





**Figure S5.** XPS survey spectrum of EG nanosheets.


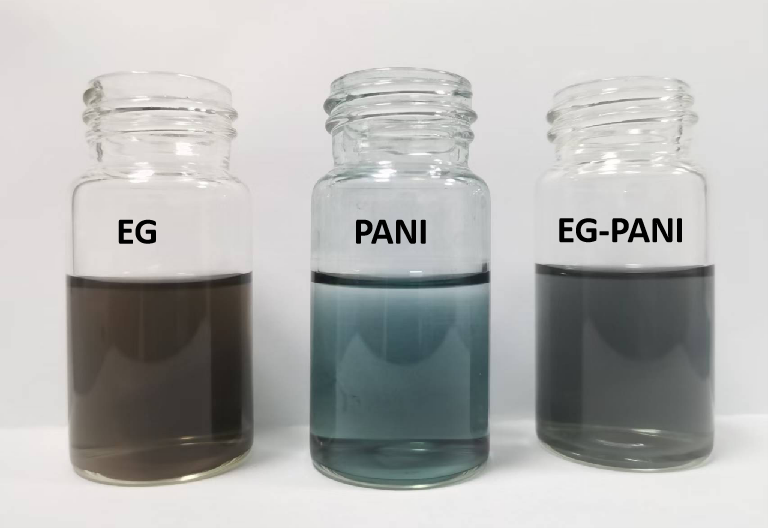


**Figure S6.** Picture of sample vials containing ethanolic dispersions of EG, PANI and EG-PANI.


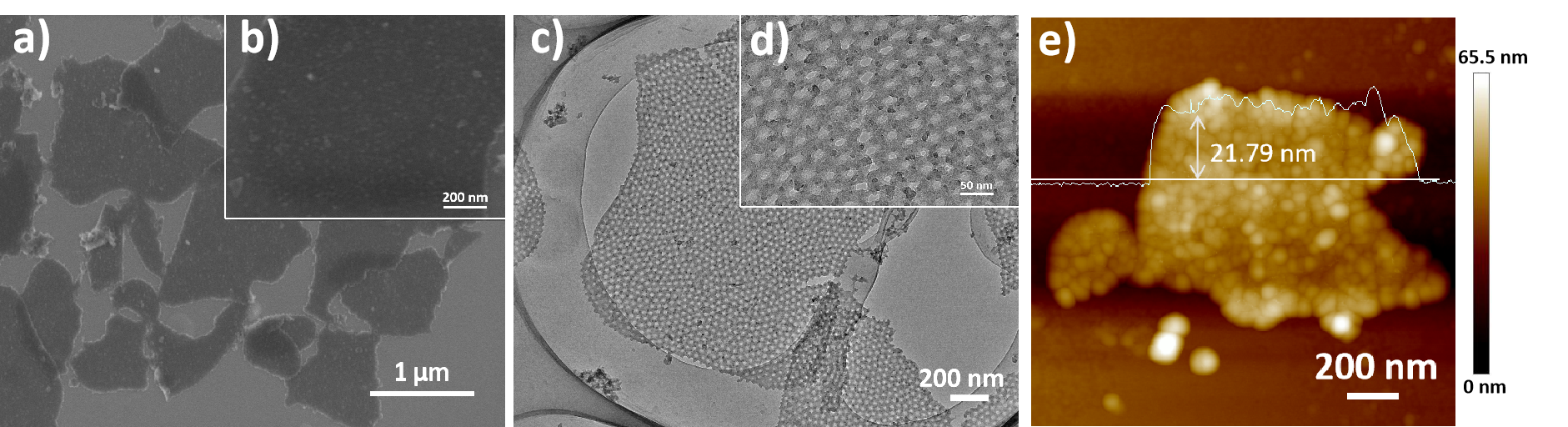


**Figure S7.** Morphological characterizations of PANI nanosheets. (a) SEM image of PANI nanosheets. (b) HR-SEM image of PANI nanosheets. (c) TEM images of PANI nanosheets. (d) The inset is the HR-TEM image of PANI nanosheet. (e) AFM image of PANI nanosheets.


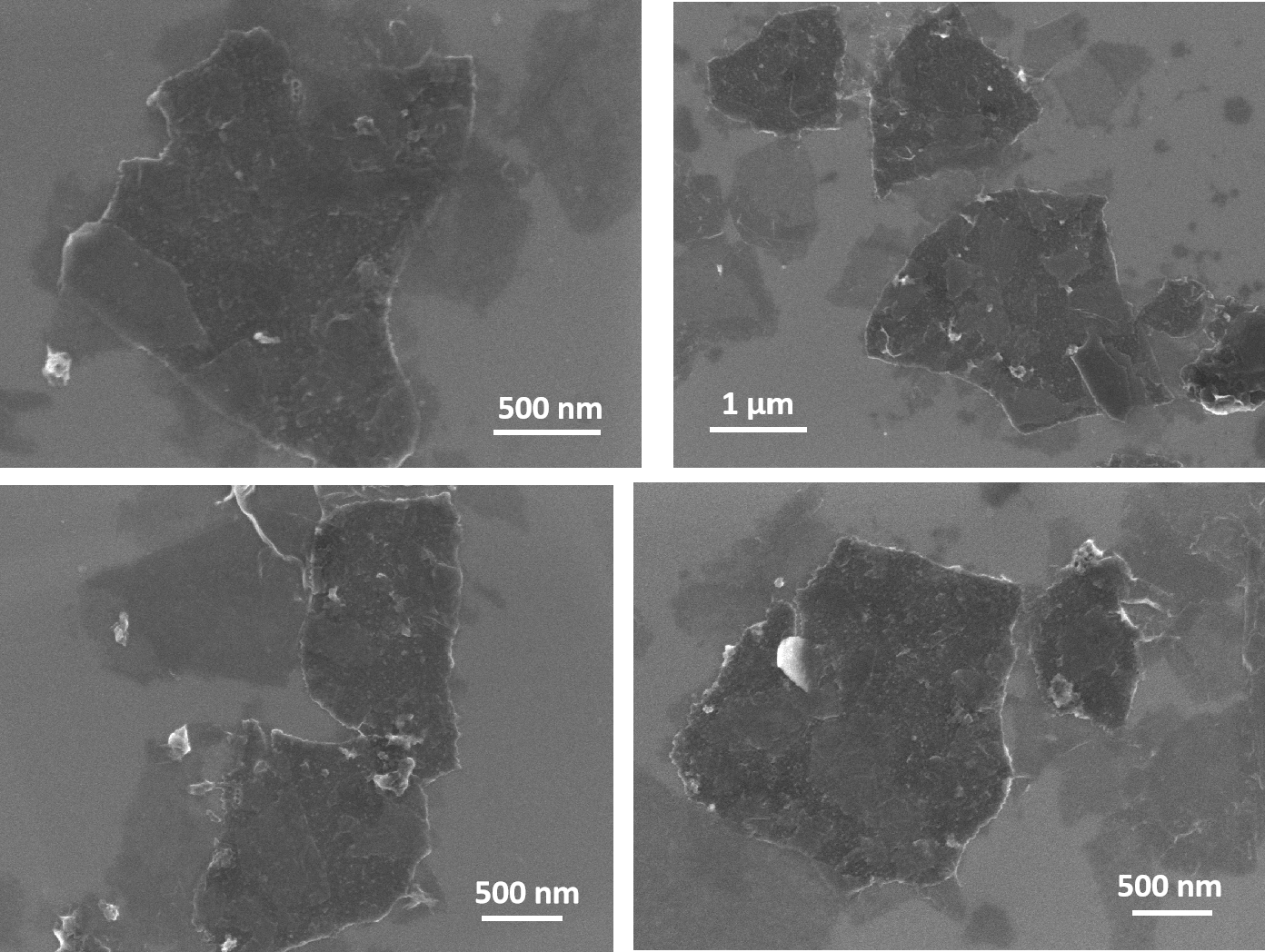


**Figure S8.** SEM images of EG-PANI.


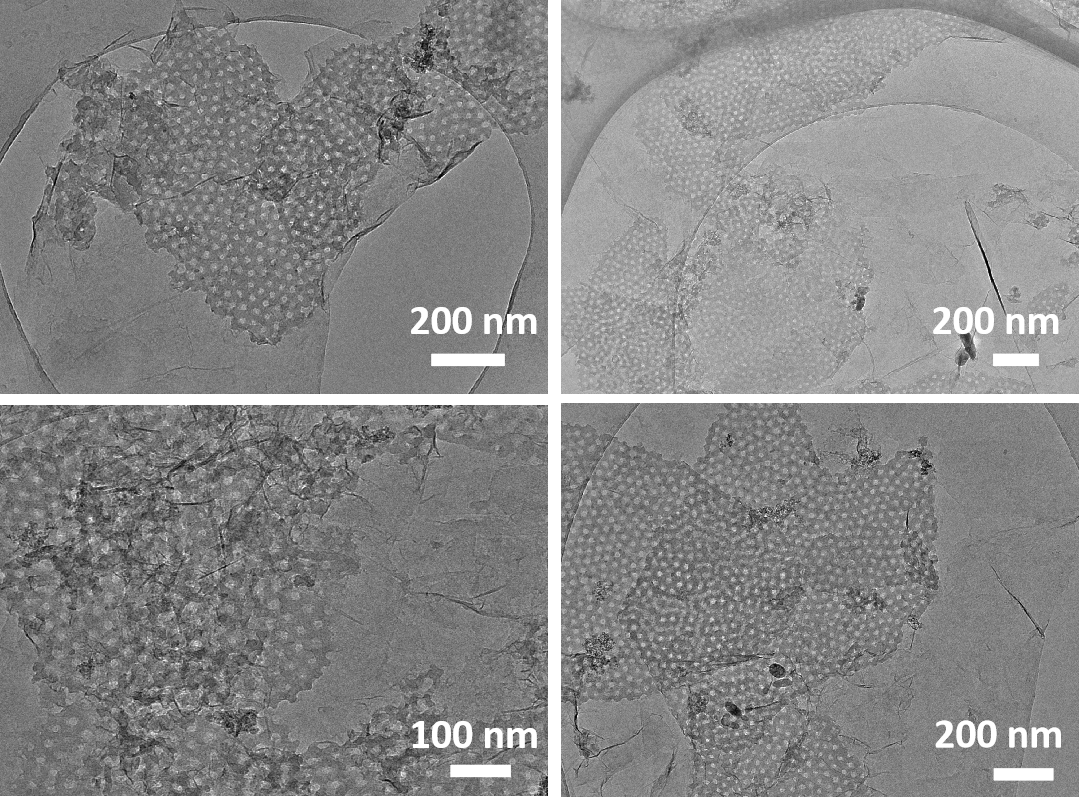


**Figure S9.** TEM images of EG-PANI.


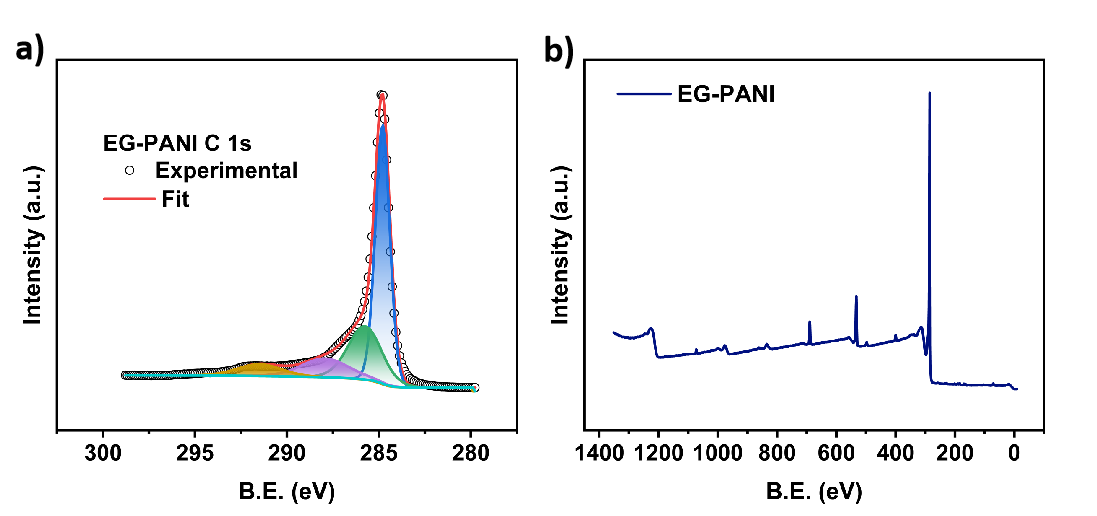


**Figure S10.** (a) High-resolution XPS of the C 1s spectrum of EG-PANI. (b) XPS spectrum of EG-PANI.


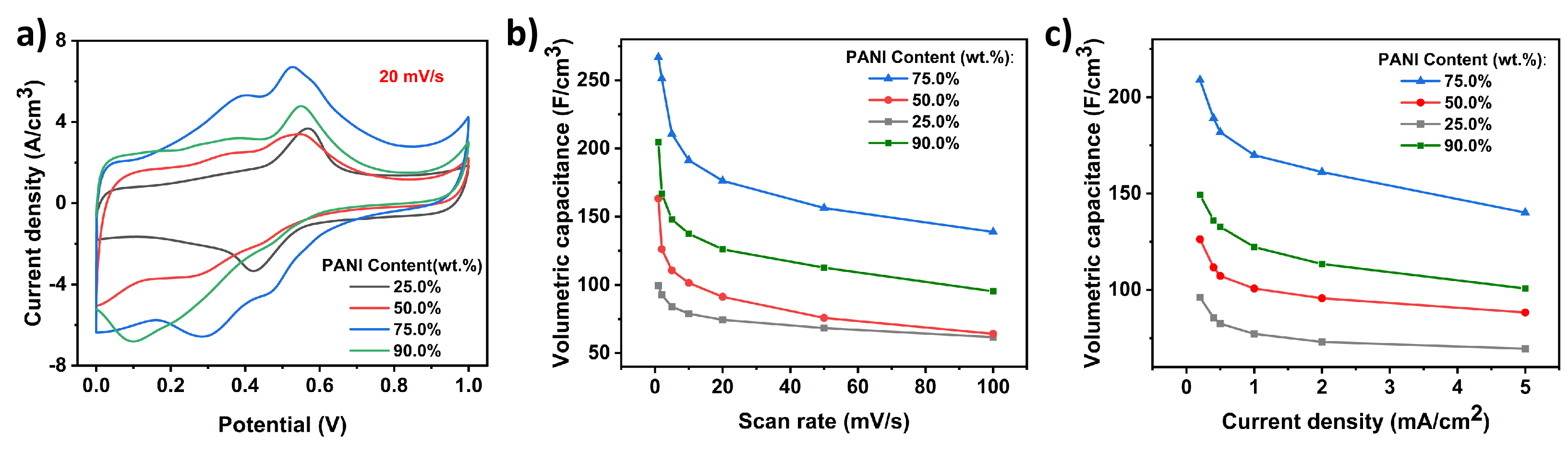


**Figure S11.** Electrochemical performance testing of EG-PANI-1, EG-PANI-2, EG-PANI-3 and EG-PANI-4. (a) CV curves, (b, c) Rate capability of EG-PANI-1, EG-PANI-2, EG-PANI-3, and EG-PANI-4.


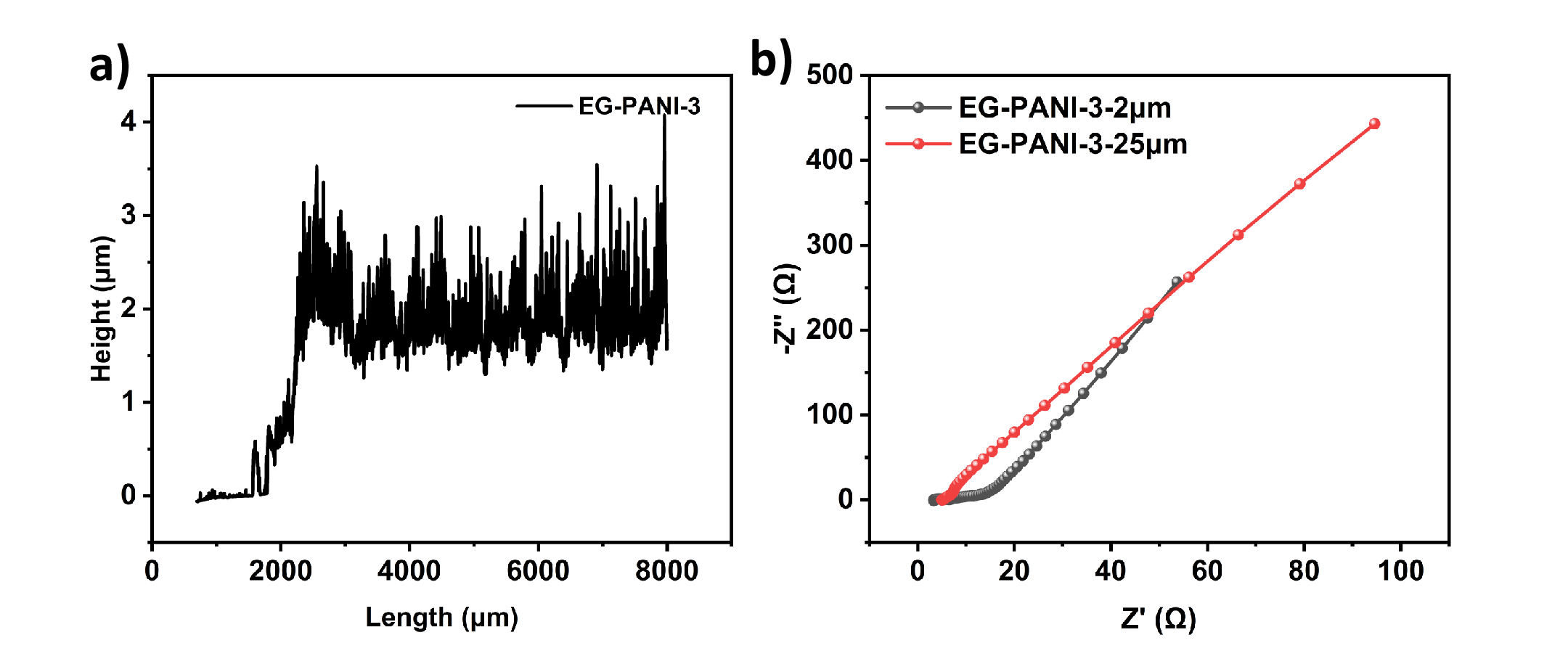


**Figure S12.** (a) Thickness of EG-PANI-3 material exploited for the three-electrode system testing. (b) Electrochemical impedance spectra of EG-PANI-3 with different thickness films (2 and 25 μm, respectively).

|  |  |
| --- | --- |
|  | 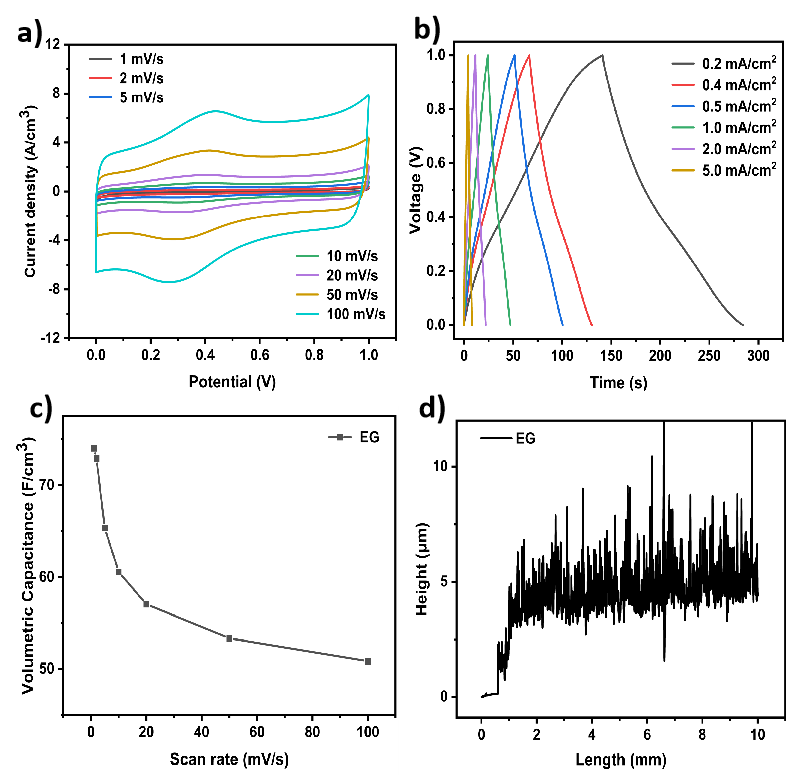 |

**Figure S13.** Electrochemical characterization of EG. (a) CV curves, (b) GCD curves of EG. (c) Rate capability of EG. (d) Thickness of EG material exploited for the three-electrode system testing.


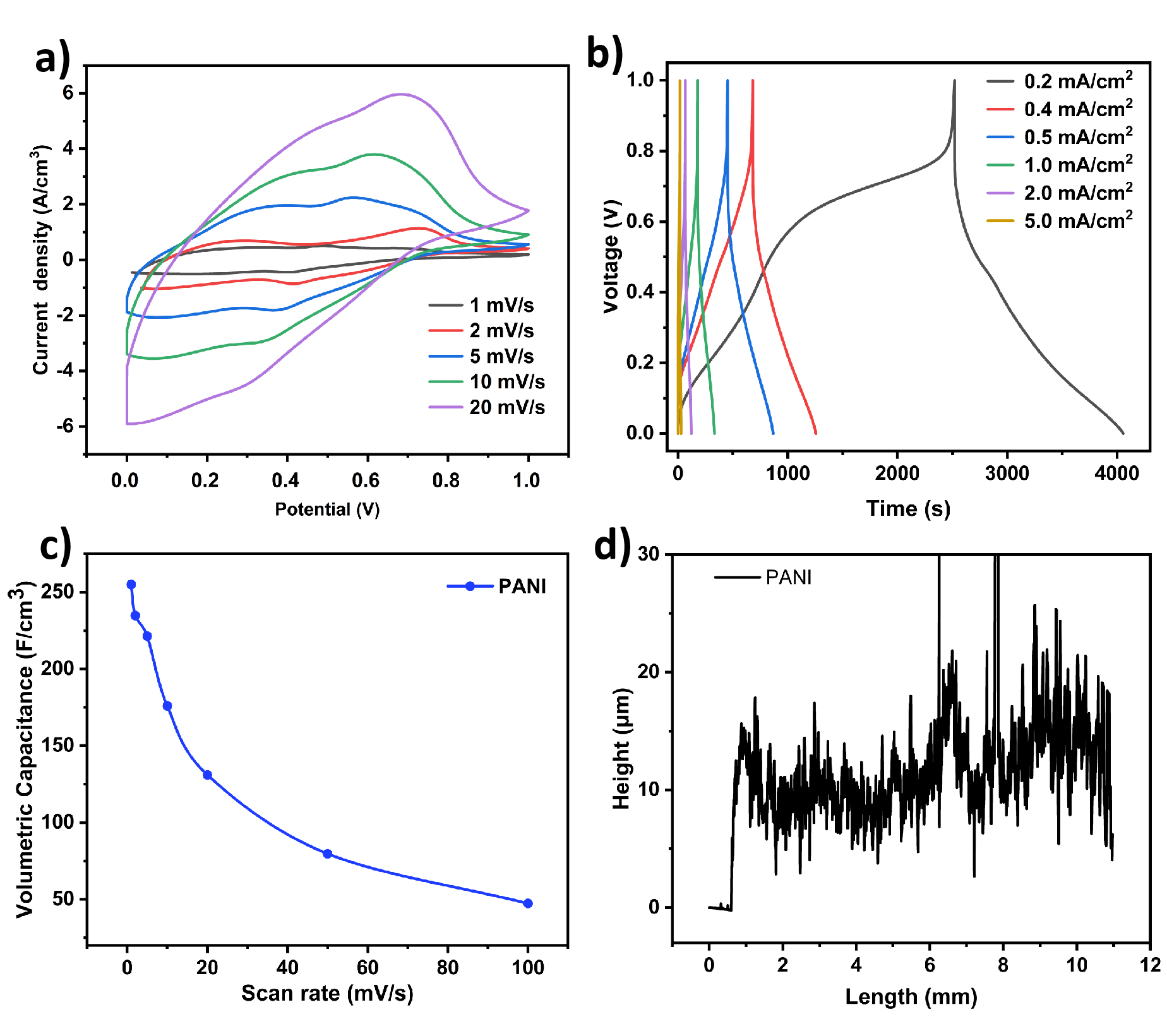


**Figure S14.** Electrochemical characterization of PANI. (a) CV curves, (b) GCD curves of PANI. (c) Rate capability of PANI. (d) Thickness of PANI material exploited for the three-electrode system testing.


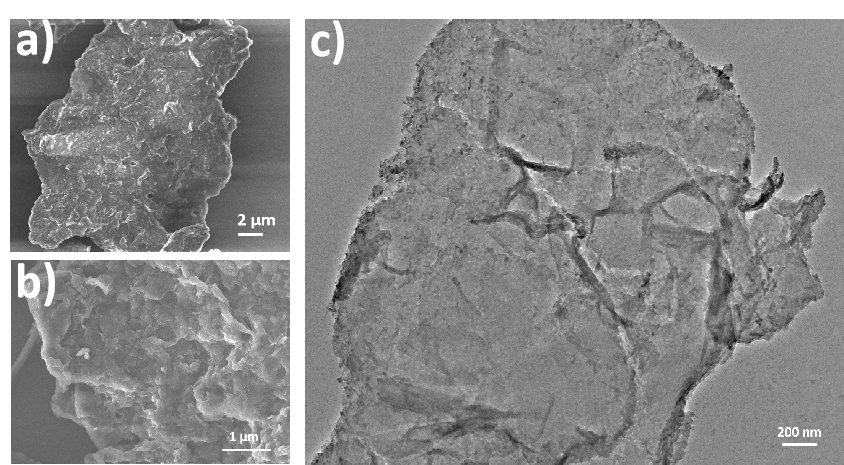


**Figure S15.** Morphological characterizations of PANI-blank nanosheets. (a, b) SEM images of PANI-blank nanosheet. (c) TEM image of PANI-blank nanosheet.


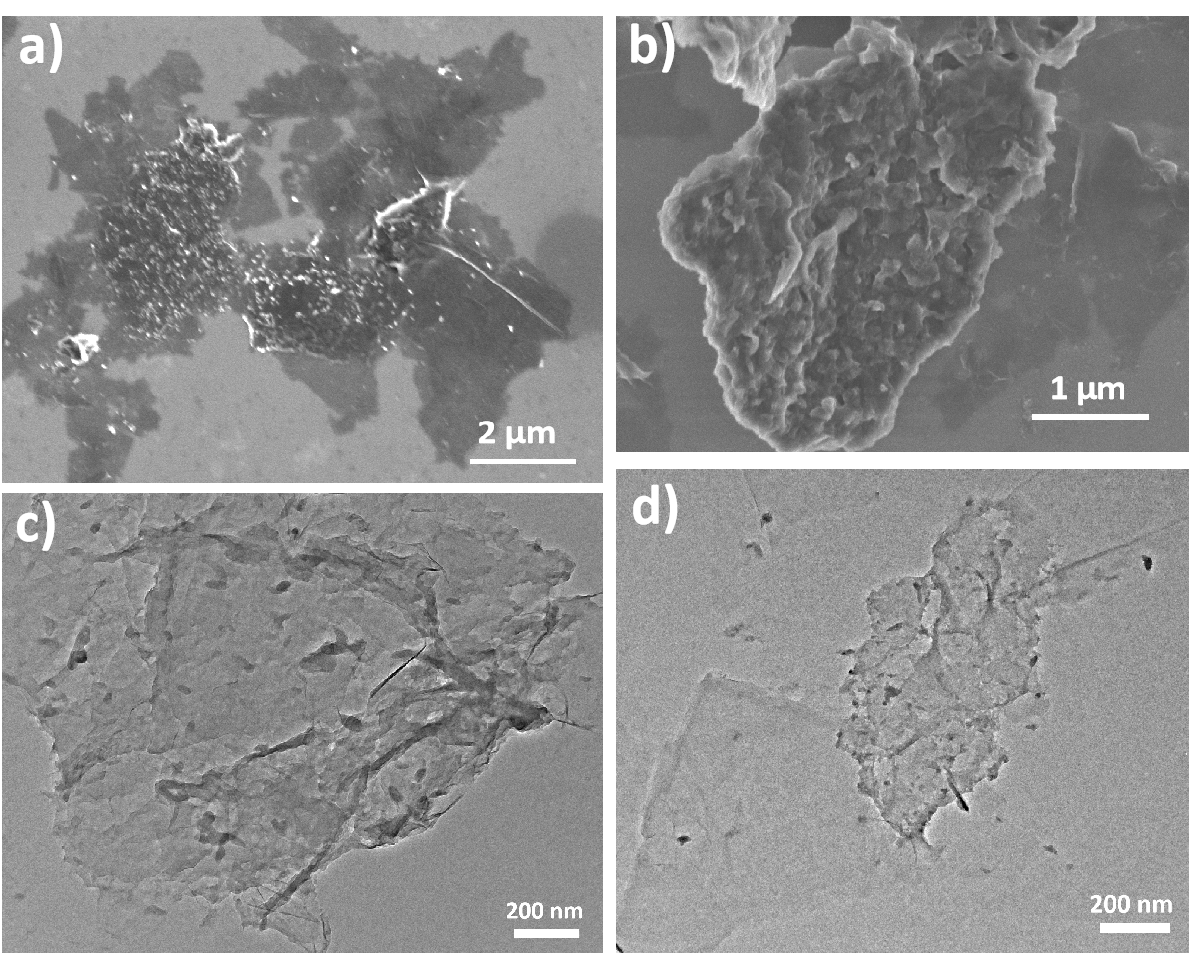


**Figure S16.** Morphological characterizations of EG-PANI-blank. (a, b) SEM images of EG-PANI-blank. (c, d) TEM images of EG-PANI-blank.


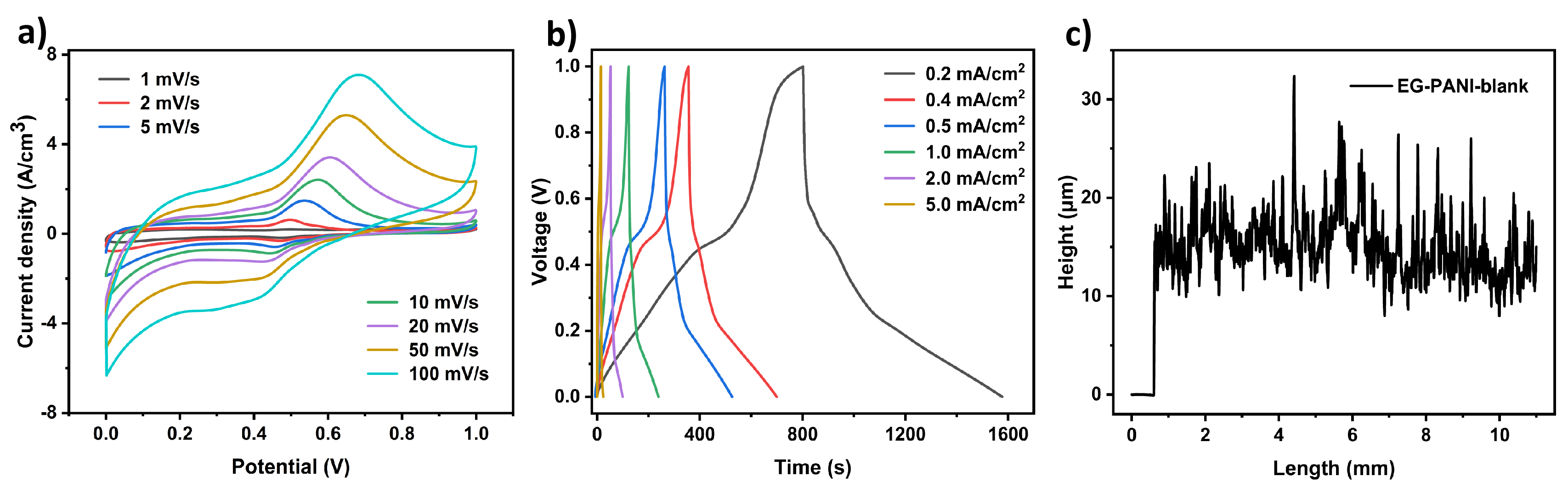


**Figure S17.** (a) CV curves, (b) GCD curves of EG-PANI-blank. (c) Thickness of EG-PANI-blank material exploited for the three-electrode system testing.


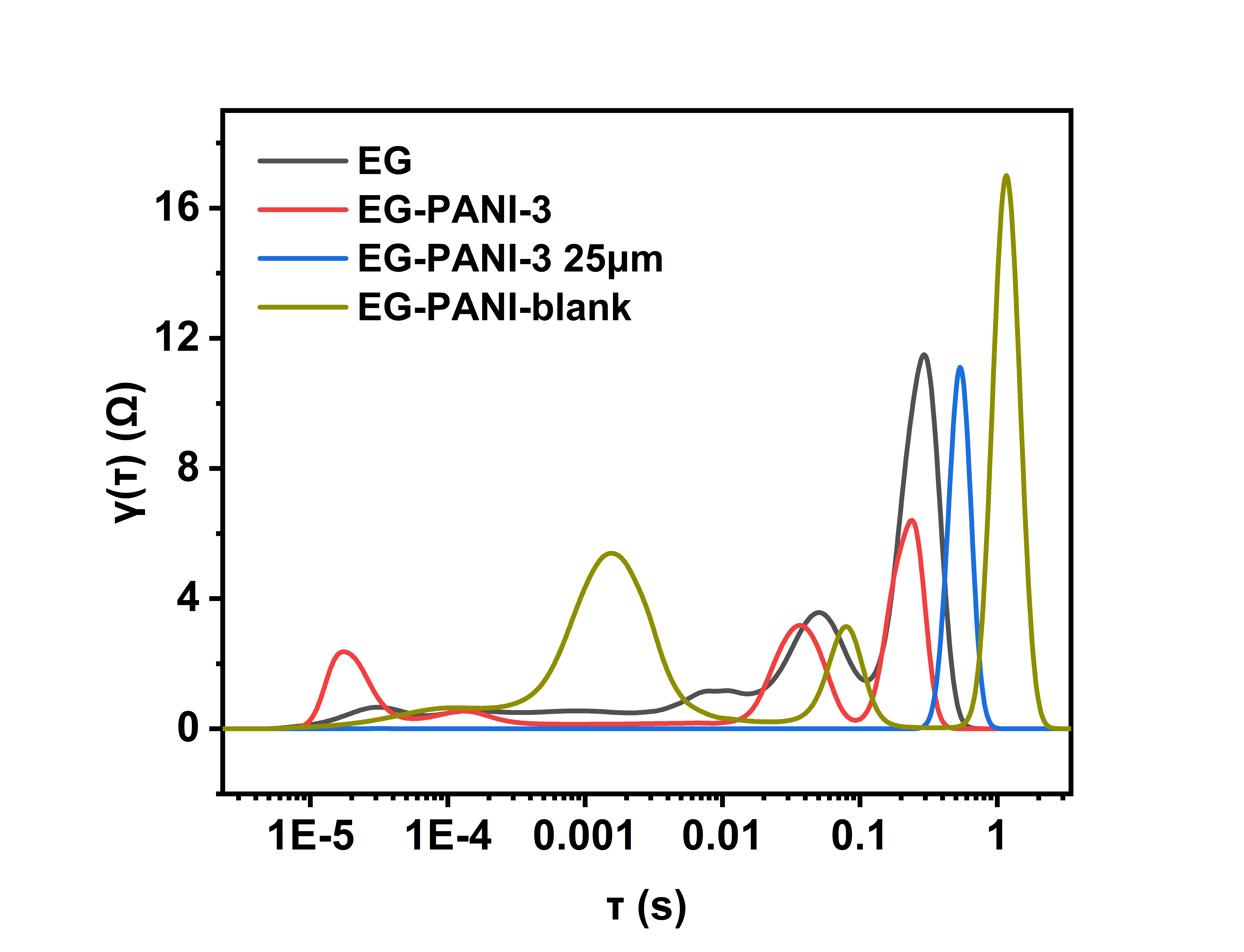


**Figure S18.** DRT of EG, EG-PANI-3, EG-PANI-blank, and EG-PANI-3-25 μm electrodes.


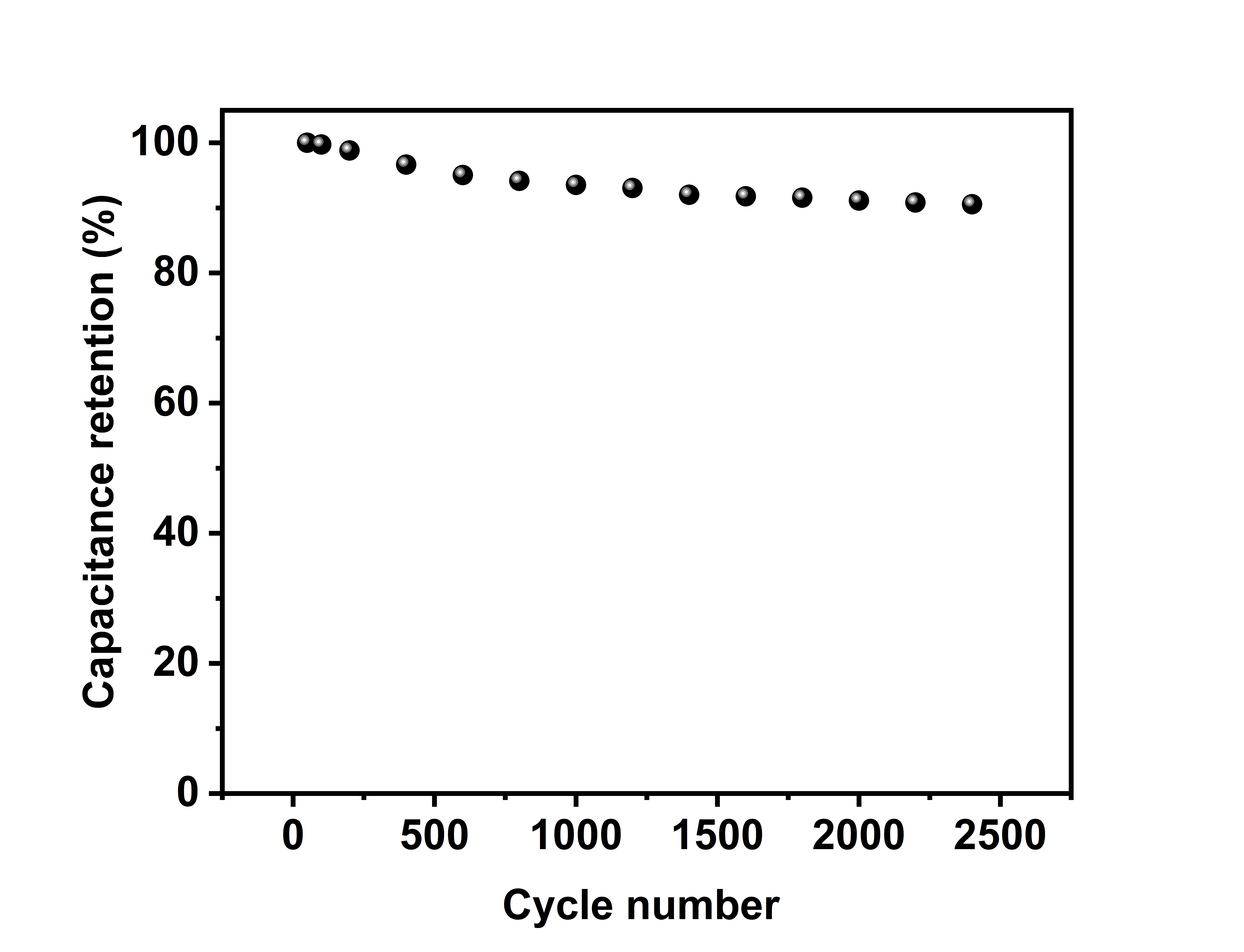


**Figure S19.** Capacitance retention of EG-PANI-3 after 2400 cycles in a three-electrode system.


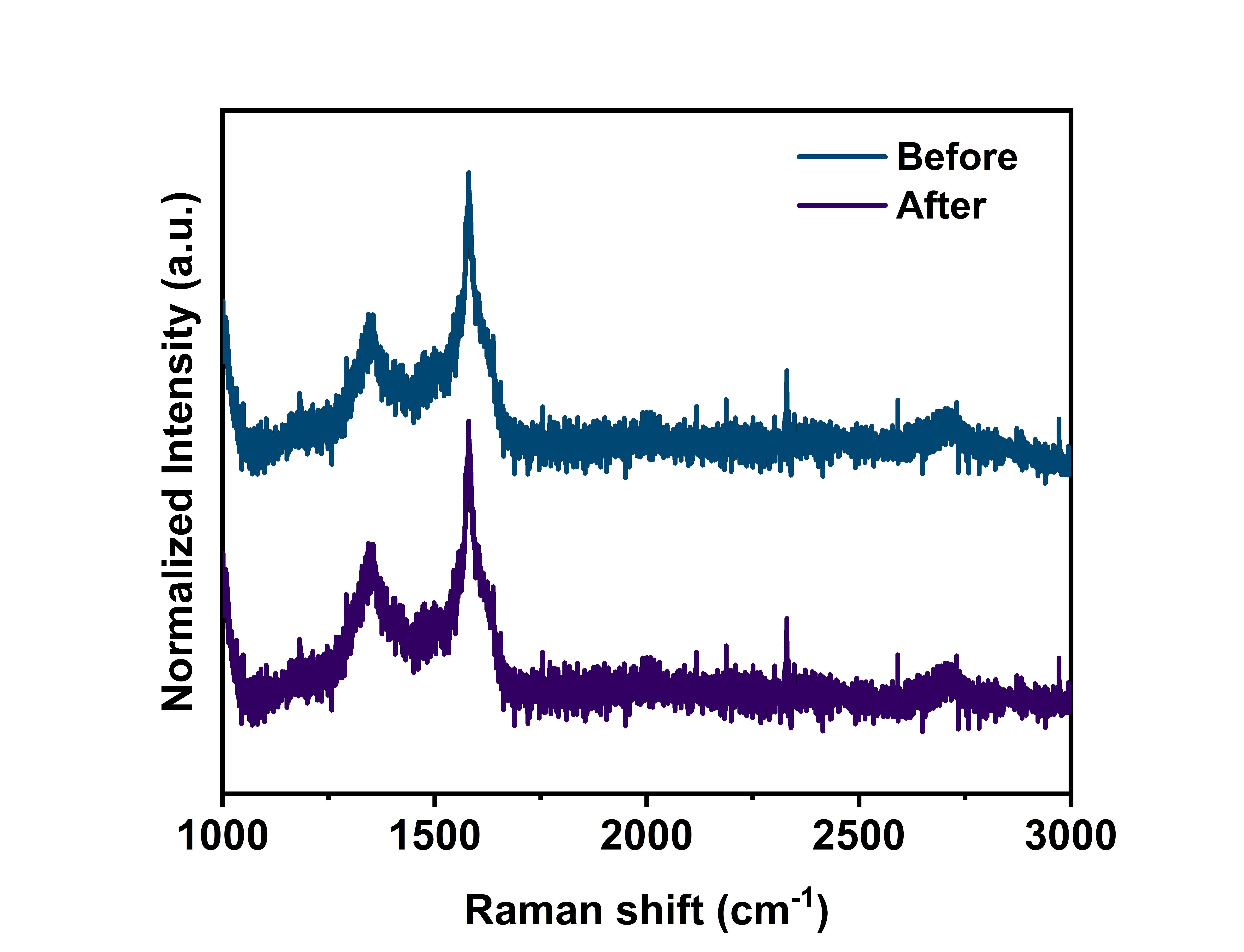


**Figure S20.** Raman spectra of EG-PANI-3 before and after 2400 cycles in a three-electrode system.


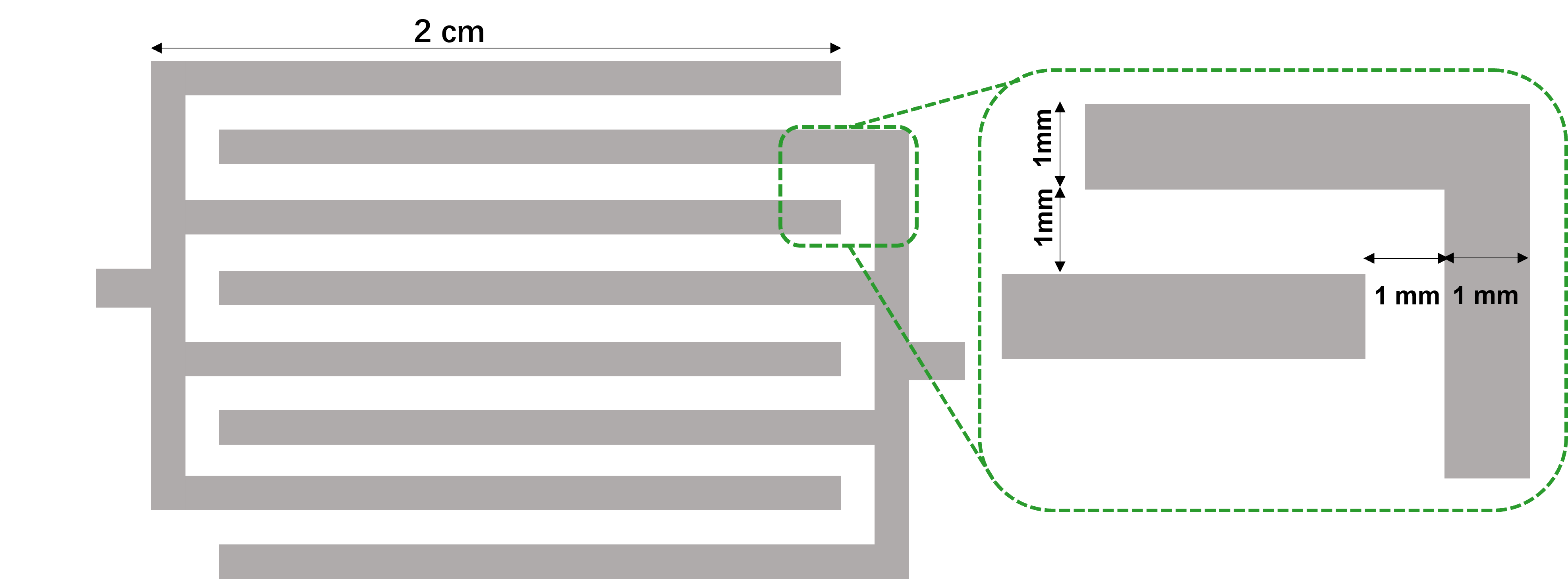


**Figure S21.** Detailed SC device geometry parameters.


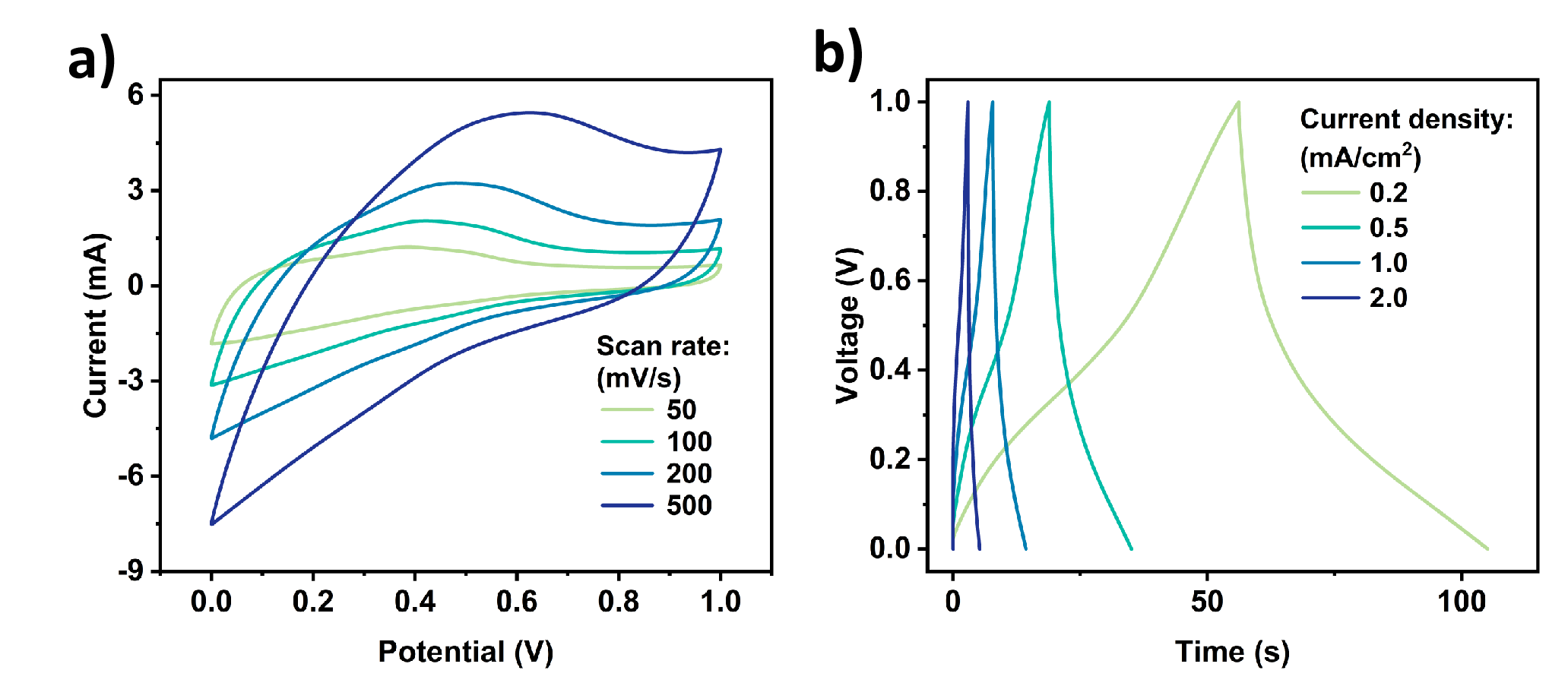


**Figure S22.** (a) CV curves of EG-PANI-SC (scan rates ranging from 50 to 500 mV/s), (b) GCD curves of EG-PANI-SC (charging/discharging current ranging from 0.2 to 2.0 mA/cm^2^).


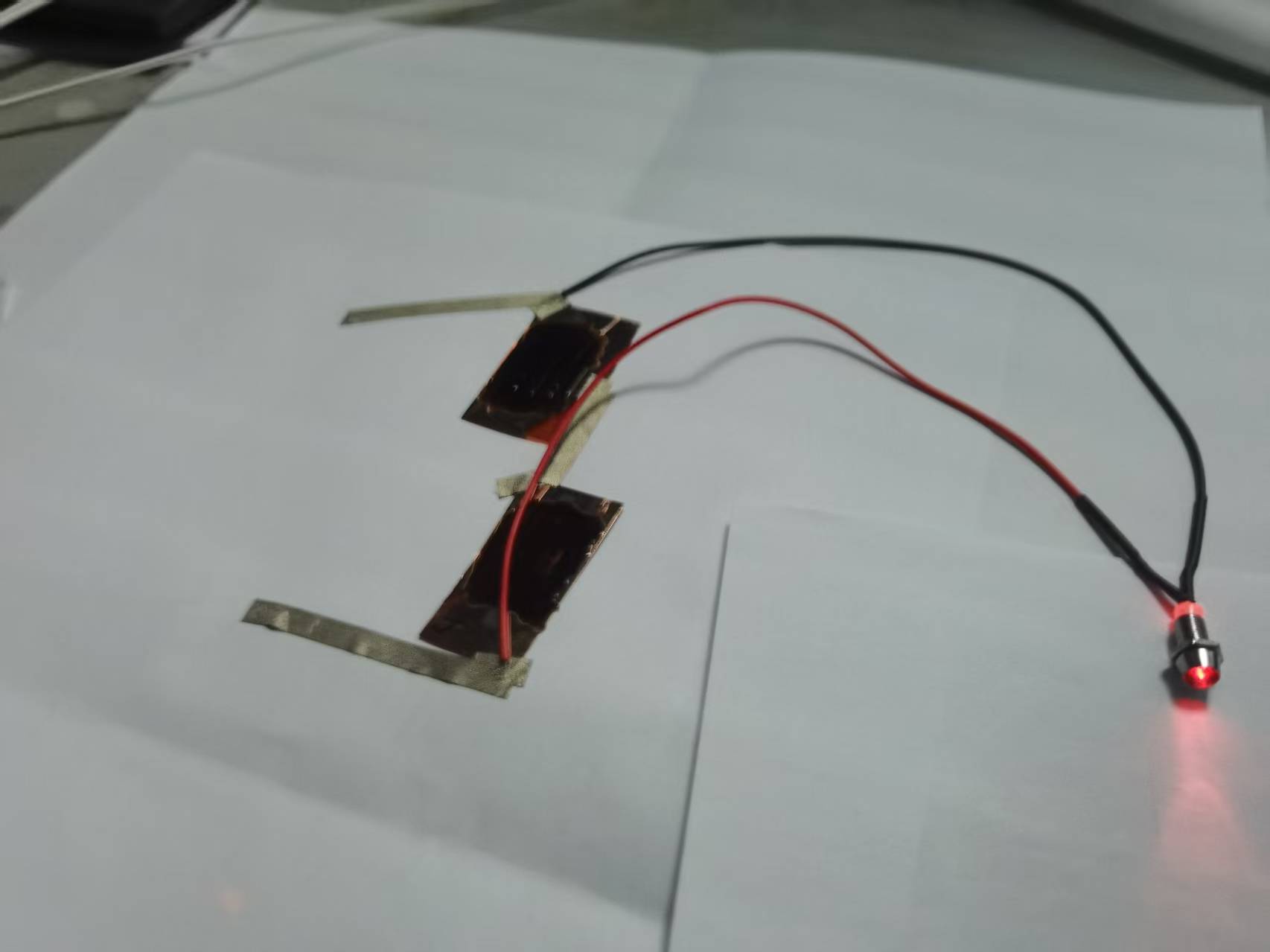


**Figure S23.** Photograph of two serially connected supercapacitors powering a red LED.


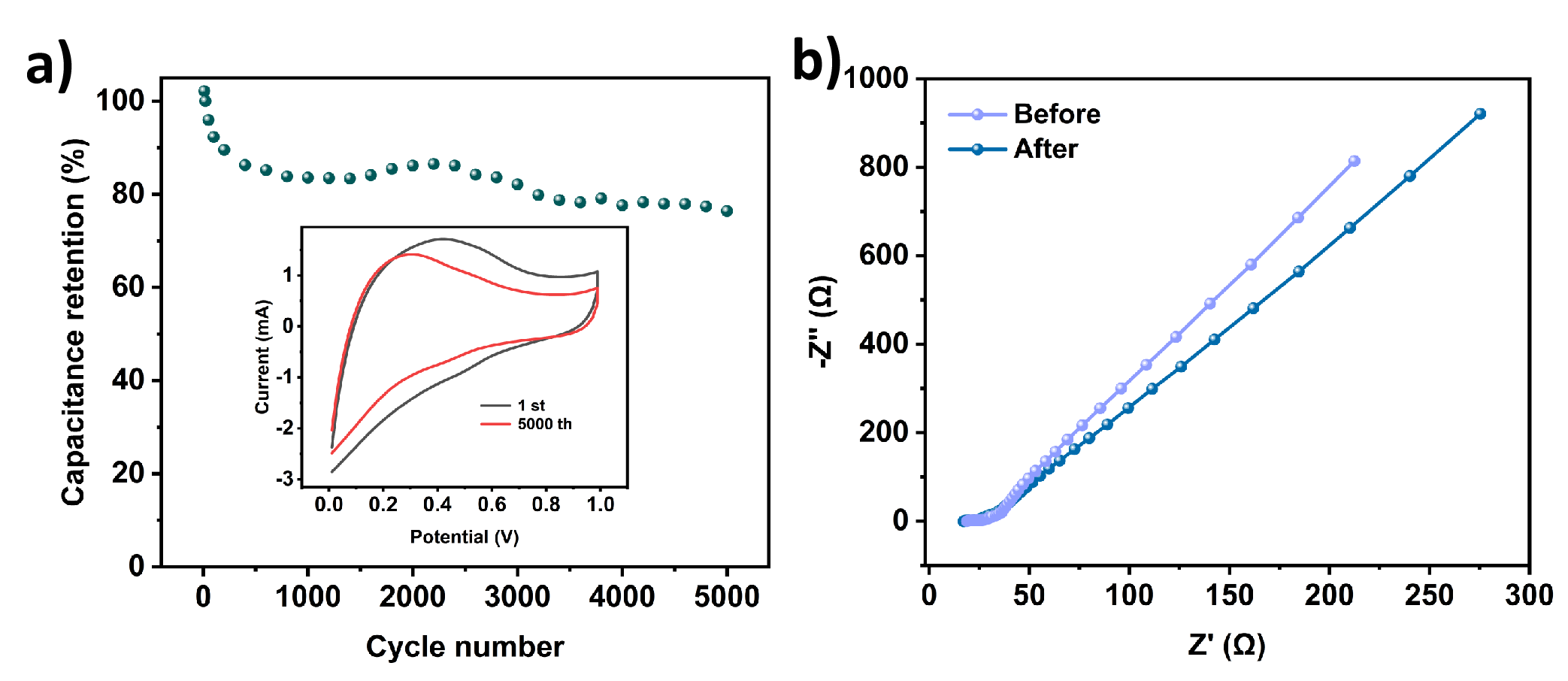


**Figure S24.** (a) Cycling stability of EG-PANI-SC over 5,000 CV cycles at a scan rate of 100 mV/s. Inset: comparison of the 1st and 5000th cycles. The device retained 76.4% of its initial capacitance after 5,000 cycles. (b) EIS of EG-PANI-SC before and after 5000 cycles.


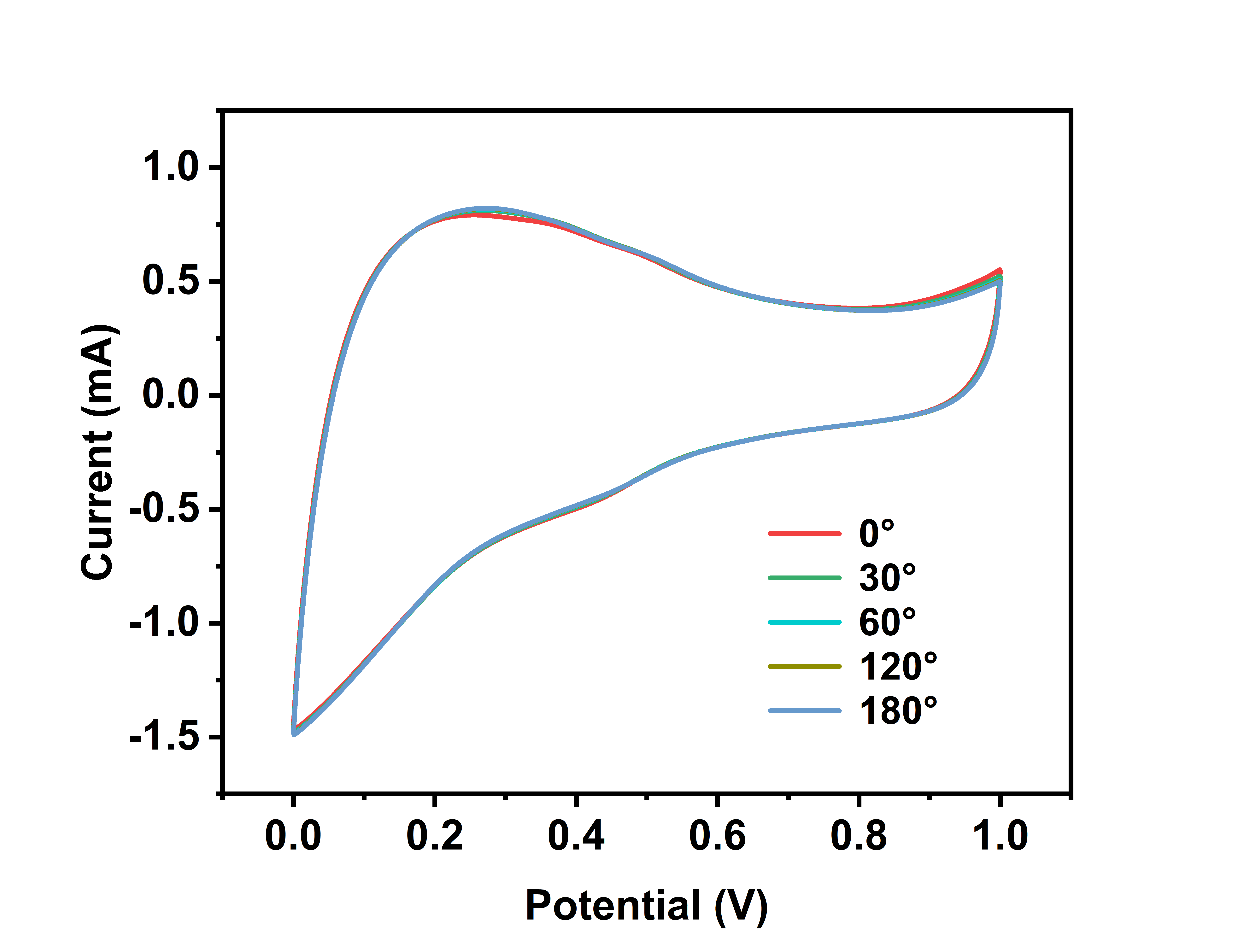


**Figure S25.** CV curves collected at different bending angles at a scan rate of 50 mV/s.

**Table S1**. Comparison of the electrochemical performance of screen-printed EG-PANI supercapacitor with previously reported graphene-PANI-based devices.

| **Ref.** | **Materials** | **Type of SC** | **Electrolyte** | **Specific capacitance** | **Cycling stability** | **Rate capability** |
| --- | --- | --- | --- | --- | --- | --- |
| [3] | Talc/PANI/PVA | Symmetric sandwich-type | 2M H_2_SO_4_/PVA | 223 mF/cm^2^  (0.5 mA/cm^2^) | 81.1% (5000 cycle) | -- |
| [4] | G-PNF30 | Symmetric flexible  film | 1M H_2_SO_4_ | 160 F/cm^3^ (0.3 A/g) | 79% (800 cycle) | 94%  (0.3-3 A/g) |
| [5] | PANI-CCG | Symmetric gel  film | -- | 532 F/cm^3^  (5 A/g) | About 85% (5000 cycle) | 96%(5-100 A/g) |
| [6] | NGP/PANI | Symmetric sandwich-type | 1M H_2_SO_4_ | 269 F/g (20 mV/s) | About 200% (1000 cycle) | 87.7% (20-100 mV/s) |
| [7] | 3D-RGO/PANI | Symmetric sandwich-type | 1M H_2_SO_4_ | 385 F/g (0.5A/g) | 88% (5000 cycle) | 94% (0.5-1A/g) |
| [8] | PAG | 3-electrode | 2M H_2_SO_4_ | 480 F/g (0.1A/g) | 70% (400 cycle) | 43.75% (0.1-1A/g) |
| [9] | Graphene-PANI | 3-electrode | 1M H_2_SO_4_ | 763 F/g (1A/g) | 82% (1000 cycle) | 64.2% (1-10A/g) |
| [10] | PANI/rGO | Symmetric sandwich-type | 1M H_2_SO_4_/PVA | 197.6 F/cm^2^ (1A/cm^2^) | 82% (3000 cycle) | 60.32% (1-20A/cm^2^) |
| [11] | PAFG(PANI+rGO) | 3-electrode | 1M H_2_SO_4_ | 1295 F/g | 88% (1500 cycle) | 47.6% (1-15A/g) |
| **This Work** | EG-PANI | 3-electrode | 0.5M H_2_SO_4_ | 424.68 F/cm^3^ (0.01mA/cm^2^) | 90.5% (2400 cycle) | 73.61% (0.2-5 mA/cm^2^) |
| **This Work** | EG-PANI | Screen-printed planar | 1M H_2_SO_4_/PVA | 16.7 mF/cm^2^ (0.01mA/cm^2^) | 76.4% (5000 cycle) | 33.8% (0.01-2 mA/cm^2^) |

**Reference**

1. S. Yang; P. Zhang; A. Shaygan Nia; X. Feng, *CCS Chemistry* **2024,** *6,* 2368.

2. Z. Liu; H. Zhang; M. Eredia; H. Qiu; W. Baaziz; O. Ersen; A. Ciesielski; M. Bonn; H.I. Wang; P. Samorì, *ACS Nano* **2019,** *13,* 9431.

3. W. Li; Z. Wang; Y. Zhu; G. Zhao; Y. Zhao; K. Zhang; A. Qin; S. Chen, *Chem. Eng. J.* **2026,** *527,* 171594.

4. Q. Wu; Y. Xu; Z. Yao; A. Liu; G. Shi, *ACS Nano* **2010,** *4,* 1963.

5. Y. Wang; X. Yang; A.G. Pandolfo; J. Ding; D. Li, *Adv. Energy Mater.* **2016,** *6,* 1600185.

6. Y. Xu; M.G. Schwab; A.J. Strudwick; I. Hennig; X. Feng; Z. Wu; K. Müllen, *Adv. Energy Mater.* **2013,** *3,* 1035.

7. Y. Meng; K. Wang; Y. Zhang; Z. Wei, *Adv. Mater.* **2013,** *25,* 6985.

8. K. Zhang; L.L. Zhang; X.S. Zhao; J. Wu, *Chem. Mater.* **2010,** *22,* 1392.

9. H.-P. Cong; X.-C. Ren; P. Wang; S.-H. Yu, *Energy Environ. Sci.* **2013,** *6,* 1185.

10. Y. Lin; H. Zhang; W. Deng; D. Zhang; N. Li; Q. Wu; C. He, *J. Power Sources* **2018,** *384,* 278.

11. Y. Liu; Y. Ma; S. Guang; F. Ke; H. Xu, *Carbon* **2015,** *83,* 79.
